# A simple methodology for the quantification of graphite in end-of-life lithium-ion batteries using thermogravimetric analysis

**DOI:** 10.1016/j.isci.2023.107782

**Published:** 2023-08-29

**Authors:** Luis Arturo Gomez-Moreno, Anna Klemettinen, Rodrigo Serna-Guerrero

**Affiliations:** 1 Department of Chemical and Metallurgical Engineering, School of Chemical Engineering, Aalto University, P.O. Box 16200, Espoo, Aalto 0076, Finland

**Keywords:** Electrochemical energy storage, Analytical method in materials science

## Abstract

A new method based on thermogravimetric analysis was developed to measure the graphite content in battery material mixture. This approach exploits the thermochemical reduction of cathodic Li-transition metal oxides with anodic graphite at elevated temperatures under an inert atmosphere. Using known composition artificial mixtures, a linear correlation between cathode mass loss and sample graphite content was observed. The method was validated using industrial black mass samples and characterized traditionally to estimate and rationalize potential error sources. Thermal degradation profiles of industrial battery waste reflected those in the artificial system, demonstrating its applicability. This work also demonstrates that thermogravimetric degradation profiles can distinguish between a cathode consisting of single or multiple Li-metal oxides. Although accuracy depends on active component mixture content and impurities, it is demonstrated that the method is useful for a fast graphite content estimation. Unlike other graphite characterization techniques, the method proposed is simple and inexpensive.

## Highlights

• New method using TGA to quantify graphite in Li-ion battery waste
• Linear correlation found between mass change and graphite content
• TGA can be used to predict type of cathode in black mass samples

## Graphical abstract

**Figure:**
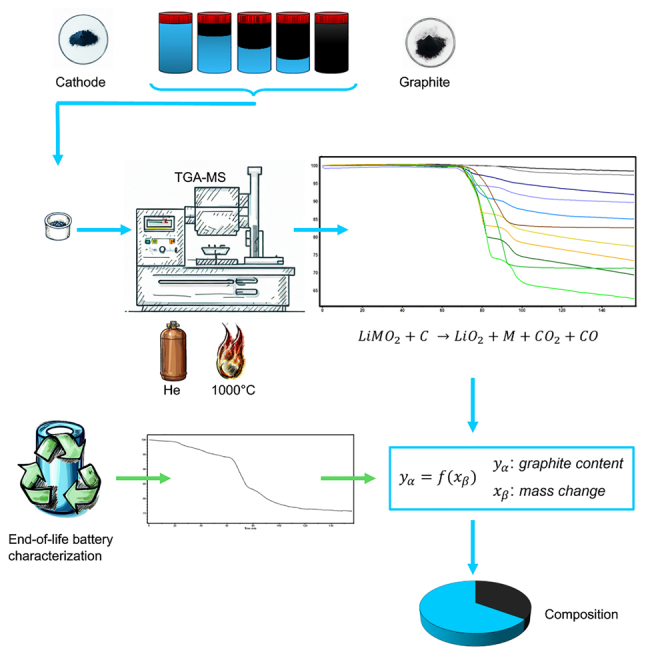

## Introduction

With the upcoming transition to decarbonize energy systems, an increase in the demand of infrastructure for renewable energy and electric vehicles is expected in the next years, requiring substantial quantities of raw materials.^1^^,^^2^^,^^3^ Li-ion batteries (LIBs) are energy storage devices that are fundamental in these ongoing decarbonization efforts^4^^,^^5^ driving a growing need for raw materials such as Li, graphite, and Co.^6^ Consequently, some of these materials are now categorized as “critical” by the European Union.^7^ This growing need for critical raw materials will represent a challenge in the near future, and alternatives to their production from virgin sources is crucial. A circular economy (CE) approach could help to increase resource efficiency and reduce waste by reintroducing end-of-life materials into the value chain.

However, the proper design of processes for LIB recycling demands a holistic CE perspective since they are associated with energy and material losses. A major challenge in the recycling of LIBs is the vast number of materials contained in a single cell. Furthermore, the diversity of applications for LIBs (e.g., electronics, power tools, electric vehicles, and medical tools) causes variations in the battery designs. This is further complicated as new materials and battery chemistries are developed and commercialized without standardization. Consequently, state-of-the-art (SoA) recycling processes recover only a few components considered economically valuable, thus not fulfilling the goals of the CE.^8^ Independently of the type of cell (pouch, cylindrical, or coin), batteries contains three main components: two electrodes and an electrolyte. In an LIB, the positive electrode (cathode) is commonly a layered or spinel Li-metal oxide (LMO) or a polyanion oxide with one or multiple alkali and two or more transition metals, i.e., LiFePO_4_ (LFP).^9^ Currently, a wide variety of different cathode chemistries are found in commercially available LIBs, each with a specific set of properties (e.g., specific energy, power, performance, safety, lifespan) that further complicates their recycling. The cathode particles are attached to Al foil as a current collector, typically using a filler to improve electrical conductivity and a polymeric binder that glues all these components together. The electrolyte is usually a Li salt dissolved in an organic solvent. Finally, the negative electrode (anode) typically uses graphite particles immobilized on Cu foils used as current collector. LIB cells also require additional components such as separators, casing, and other plastics. The chemical composition of batteries plays a key role in determining how they are recycled, as different chemistries and elements may require different conditions to extract the valuable materials.^10^^,^^11^^,^^12^ It is thus fundamental to correctly identify the composition of materials produced throughout the recycling processes for their control and optimization. The composition of metals such as Co, Ni, Mn, Cu, and Al in the battery waste streams are relatively easy to quantify. The use of techniques such as X-ray diffraction (XRD), inductively coupled plasma (ICP) spectroscopy, scanning electron microscopy (SEM), and energy-dispersive X-ray spectroscopy (EDS) for battery waste characterization can be found in the published literature for such purpose.^13^^,^^14^^,^^15^

To better address the goals of the CE and the forecasted increase in raw material demands, the recovery of graphite from LIBs has recently caught the attention of scientist and practitioners.^15^^,^^16^^,^^17^^,^^18^^,^^19^^,^^20^^,^^21^ However, carbon-containing species represent a special case for characterization, as the distinction between total organic carbon (TOC), total inorganic carbon (TIC), and elemental carbon (EC) is not trivial. In the existing literature on characterization of LIB active components mixture, i.e., the so-called “black mass (BM)”, graphite anode content is not usually quantified but rather total carbon (TC) is reported, including TIC and TOC. Various forms of carbon can be found in the battery waste as part of its composition or as impurities of the raw materials used in their production. Due to its relevance in various industries and fields, there are multiple methods available for the characterization of carbon-containing species. Some common techniques include Raman spectroscopy,^16^ Fourier transform infrared (FTIR) spectroscopy,^22^ combustion analysis,^23^ and X-ray photoelectron spectroscopy (XPS).^24^ Although techniques such as ICP spectroscopy^24^ and thermogravimetric analysis (TGA)^25^ are not specifically designed to measure carbon-containing species, they can be used to infer the carbon content of a sample. The most common method to measure TC is combustion analysis in which the carbon in a sample reacts with pure oxygen gas while the CO_2_ and CO products are monitored with infrared absorption spectroscopy to determine the carbon content.^26^ Additionally, gas chromatography and thermal conductivity detectors after combustion can be used to obtain a precise calculation of carbon and other elements such as hydrogen and sulfur.^27^ Nevertheless, these methods do not discriminate between the forms of carbon present in the sample and thus require sample preparation methods that are currently expensive, time consuming, and potentially hazardous.^26^ For example, a typical sample preparation method consists of leaching the material with HCl to remove carbonates, followed by heating at 530°C to eliminate TOC.^28^ The remaining sample is expected to have only EC in the form of graphite. The sample is then placed into a combustion furnace and purged with an inert gas to remove air. Pure O_2_ is used as the combustion agent, and the sample is heated, causing the graphitic carbon to oxidize to CO_2_. The CO_2_ is collected for analysis using an infrared detector or gas chromatography and quantified to relate it to the graphitic carbon content using the stoichiometry of the reaction.^28^ It should be noted that these degradation-based techniques were not developed considered the potential reactivity between graphite and LMOs as found in the BM.

Some techniques capable of differentiating the atomic structure and energy spectrum between amorphous carbon and graphite can be used to distinguish between graphitic carbon and other carbon species, including XRD, Raman spectroscopy, SEM, and transmission electron microscopy. Semi-quantitative analysis can also be performed with electron microscopy based on the morphology and structure of the carbon species.^29^^,^^30^^,^^31^ These techniques offer different advantages in terms of accuracy, speed, and cost and can be used in combination to obtain a more comprehensive characterization of the sample. Following these procedures, the quantification of graphite in battery waste is inexact and difficult. In LIB recycling processes, a simple graphite characterization is needed since it affects the efficiency of operations such as flotation,^17^ pyrometallurgy,^32^ or hydrometallurgy.^33^

The present work proposes a simple and fast methodology to estimate the graphite content in BM samples using TGA supported by evolved gas analysis (TGA-EGA). The method is based on the premise that cathode compounds in BM can be reduced to elemental metals at high temperatures in the presence of a suitable reducing agent. In an inert atmosphere, a mass loss detected by TGA will thus be associated with the release of CO and CO_2_ that is correlated to the graphite content. This idea is in direct contrast to previously published studies using TGA under flowing air,^34^^,^^35^^,^^36^ where uncontrolled combustion reactions take place, making it difficult to correlate mass changes with the BM composition. To develop the novel approach hereby proposed, TGA-EGA results were performed on artificial mixtures of graphite and cathode materials, i.e., Li(Ni_0.33_Mn_0.33_Co_0.33_)O_2_ (NMC) or LiCoO_2_ (LCO) at known compositions to produce standardized correlation functions. The obtained results are finally validated in the analysis of industrially produced BM samples of unknown composition, demonstrating its applicability in real-life context.

## Results and discussion

### Thermal characterization of artificial BM

**Figure 1:**
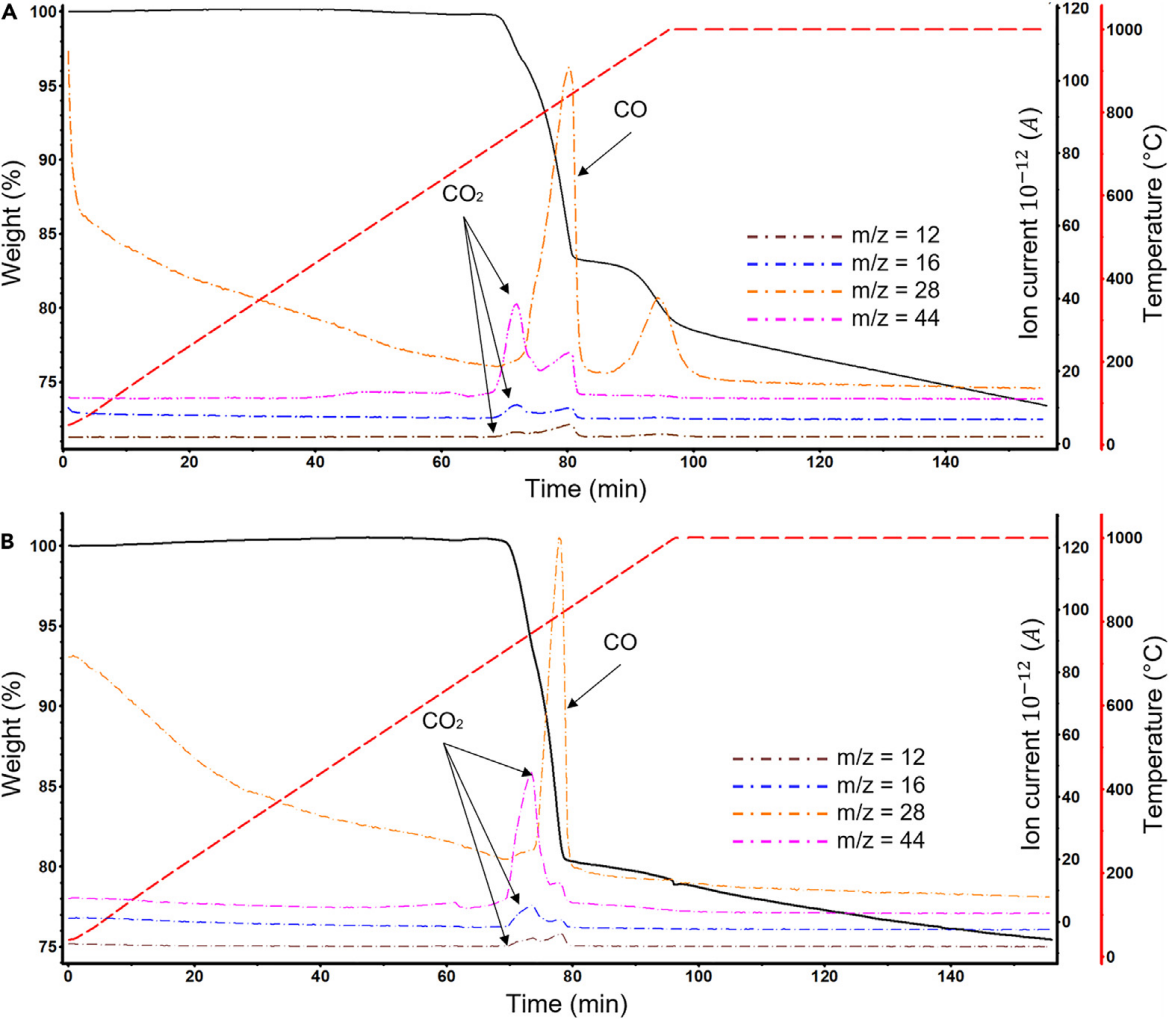
TGA-EGA of artificial black mass with 50 wt (A and B) % graphite and 50 wt. % NMC (A) and 50 wt. % graphite and 50 wt. % LCO (B).

The TGA-EGA decomposition profile of an exemplary artificial BM (1:1 graphite to NMC ratio) is shown in Figure 1A. As seen, the thermal decomposition presented three characteristic regions. During the first region, there was no visible mass loss below 684°C. The second region has a distinctive shape with three different steps of mass loss. The first mass loss was associated with ion current values m/z = 12, 16, and 44, all characteristic of CO_2_.^37^ This is followed by a strong signal of CO evolution (m/z = 28) along with a minor release of CO_2_. The third region presents a comparatively slower mass loss up to ca. 900°C, and only CO evolution was detected in a meaningful quantity. Since the thermal decomposition occurred under an inert environment, the mass loss can only be explained as a result of the chemical reduction of the cathode materials by graphite. According to the work by Babanejad et al.,^38^ three types of reactions occur during this thermal reduction:

(Equation 1) $$Li({Ni}_{0.33}{Mn}_{0.33}{Co}_{0.33}O_{2}+1.6C\;\to 0.33Ni+0.33Mn+0.33Co+Li+1.2CO+0.4{CO}_{2}$$

(Equation 2) $$Li({Ni}_{0.33}{Mn}_{0.33}{Co}_{0.33}O_{2}+1.2C\;\to \;0.33Ni+0.33Mn+0.33Co+{0.5Li}_{2}O+0.9CO+0.3{CO}_{2}$$

(Equation 3) $$Li({Ni}_{0.33}{Mn}_{0.33}{Co}_{0.33}O_{2}+0.9C\;\to \;0.33Ni+0.33MnO+0.33Co+{0.5Li}_{2}O+0.7CO+0.2{CO}_{2}$$

The reduction of Ni and Co from NMC with graphite is theoretically possible starting at 440°C and 500°C, respectively (See Figure S13 in supplementary information). Mn reduction is more difficult due to its standard Gibbs free energy of formation (-Δ G°) making its oxide form relatively stable.^39^ However, it can be reduced with C at higher temperatures (>550°C). Theoretically, Li_2_O is the most difficult oxide phase in NMC to reduce, as it has the lowest Gibbs energy from the oxides present.^40^ Under the conditions used in this study, only Ni, Co, and Mn are reduced while Li likely remained in its oxide form. This allows the proposal of an overall chemical reaction (Equation 4) that is compatible with the TGA profile in Figure 1A. The thermal decomposition begins at 450°C with Ni and is followed by Co at 500°C and finally Mn at 800°C, corresponding well with the three decomposition steps in Region II. The final isothermal phase shows a minor, although continuous, mass loss. Although no more CO or CO_2_ ion current signals were observed beyond this temperature, it is possible that further reduction of Mn is occurring. Admittedly, the extent of Mn reduction is uncertain, but, for the sake of simplicity, we can assume that all transition metals are reduced and equimolar amounts of CO and CO_2_ are produced, following Equation 4:

(Equation 4) $$Li\left({Ni}_{0.33}{Co}_{0.33}{Mn}_{0.33}\right)O_{2}+C\;\to 0.5CO_{2}+0.5CO+0.33Ni+0.33Mn+0.33\;Co+0.5{Li}_{2}O$$

Stoichiometrically, this would require an equal number of moles of cathode and graphite species, resulting in a maximum theoretical mass loss of 40.6 wt. % of the total mixture based on the reaction involving 1 mol of NMC and 1 mol of graphite. For the LCO cathode, the value is 40.1 wt. % of the total mixture, as both cathodes have similar molecular weight.

A similar behavior occurs in the presence of LCO cathode, as shown in Figure 1B. The LCO-containing artificial BM initially experiences a minor mass loss below 550°C, attributed to impurities in the reagents. The reduction reaction starts at 683°C, a similar temperature as in the NMC cathode. The main difference when compared to NMC is the profile of the mass loss curve. In LCO, the mass loss occurs in a single step, with a final inflection point at 938°C. This difference in behavior is likely due to the multiple metals in NMC as opposed to LCO, which only contains Li and Co. Additionally, with both cathode materials, CO_2_ is released first, followed by CO. The reduction of the LCO cathode has been reported previously by various other authors^41^^,^^42^^,^^43^^,^^44^^,^^45^ under different temperature and atmospheric conditions. The proposed mechanism of thermal reduction for LCO-containing BM based on TGA-EGA results is shown in Equation 5.

(Equation 5) $$LiCoO_{2}+C\;\to Co+0.5{Li}_{2}O+0.5CO_{2}+0.5CO$$

As seen in Figure 1, with both NMC and LCO mixtures containing 50% graphite, the mass change attributed to cathode reduction was approximately 25%. This is likely because in both cathode materials the relation between reducible metals (i.e., Ni, Mn, and Co for NMC; Co for LCO) and oxygen is similar: 0.59 for NMC and 0.6 for LCO. Under an inert environment, the oxidation of graphite and subsequent reduction of metallic species depend on the amount of O available from the cathode compound.

**Figure 2:**
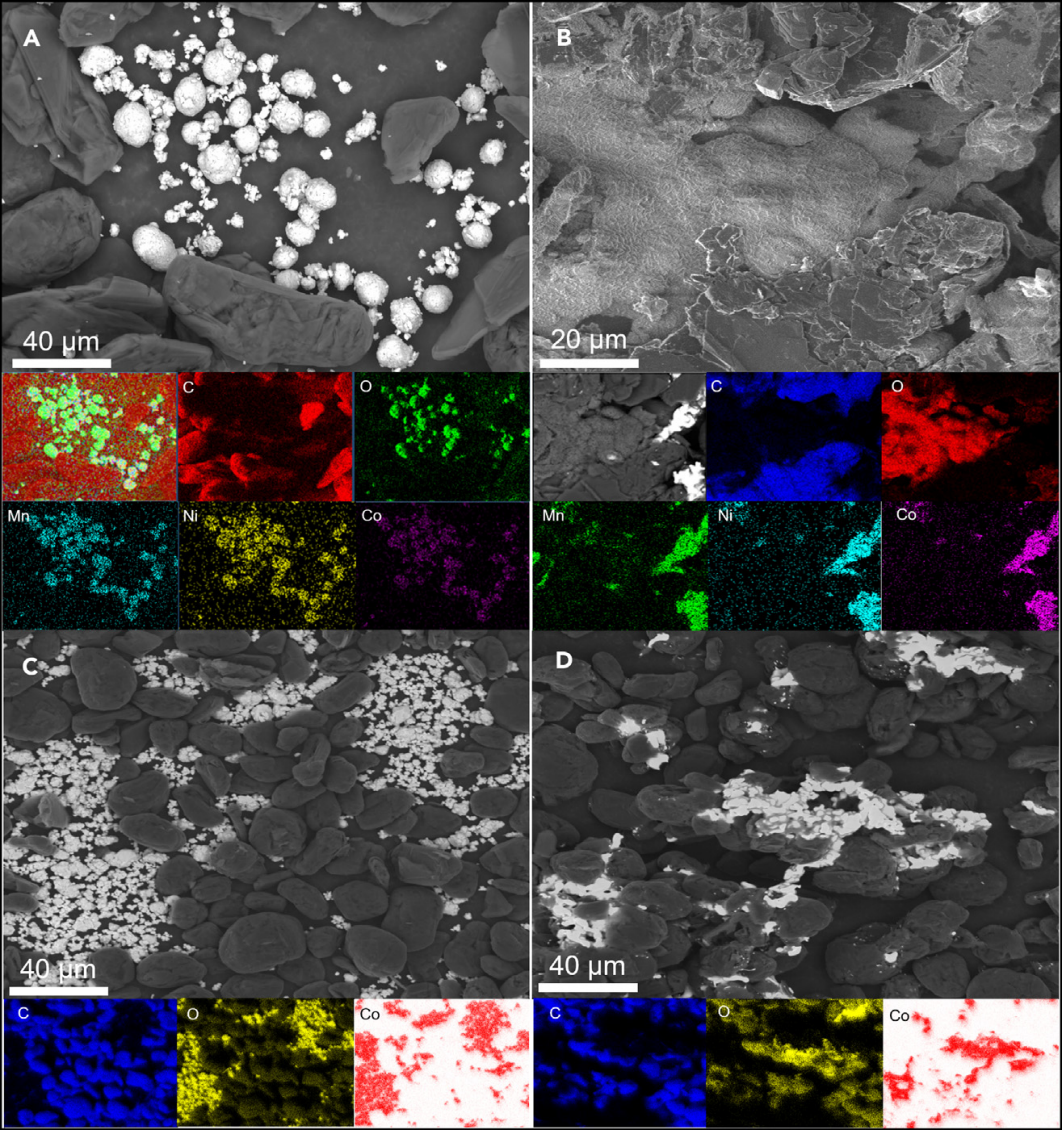
Detailed imaging and elemental mapping (EDS mode) of artificial BM (A) Bulk BM with 50 wt. % graphite and 50 wt. % NMC, (B) BM with 50 wt. % graphite and 50 wt. % NMC after thermal treatment at tube furnace, (C) bulk BM with 50 wt. % graphite and 50 wt. % LCO, and (D) BM with 50 wt. % graphite and 50 wt. % LCO after thermal treatment at tube furnace.

The NMC-graphite chemical reaction in Equation 4 occurs at a higher temperature than the melting point of the pure metallic species contained in the cathode.^46^ It is thus likely that an alloy of such metals is formed after the thermal treatment. This was confirmed by the SEM-EDS analysis of the TGA products from the artificial BM sample, shown in Figure 2B. The SEM image also shows that the BM components have lost their original morphology, as shown in Figure 2A which depicts the sample prior to undergoing thermal treatment, where graphite and NMC show their original morphology. However, three distinct phases are still recognizable: a metallic alloy; residual graphite; and an undefined continuous phase with a high O_2_ content. The EDS elemental analysis corroborated that the alloy is composed of Ni, Co, and Mn. In the case of NMC, the high O_2_ compound did not appear to be associated with either transition metals or graphite, suggesting it is some form of Li oxide (e.g., Li_2_O). The topology of the material indicates that particles have melted and then resolidified, as the cathode particles lost their spheroidal shape. Unexpectedly, graphite also lost its individual particulate form, appearing as a continuous phase, mixed with the Li oxide and the metal alloy. In contrast to NMC, LCO has a melting point of 1,100°C,^46^ and so, any chemical reactions occur only in the solid phase. Figure 2C shows an artificial BM sample using an LCO cathode. Initially, the LCO cathode exhibits morphology similar to NMC, with a spheroidal shape. As observed in Figure 2D, the changes in morphology for LCO after thermal treatment suggest that the cathode material experienced a series of complex transformations. The new morphology appears to be formed by an inner layer of metallic Co surrounded by an intermediate phase where oxygen and carbon overlap, suggesting the presence of Li_2_CO_3_ species. Indeed, Li_2_CO_3_ has been identified by other authors^38^ as an intermediate species during the reduction of cathode materials.

### Graphite estimation methodology using TGA-EGA analysis

**Figure 3:**
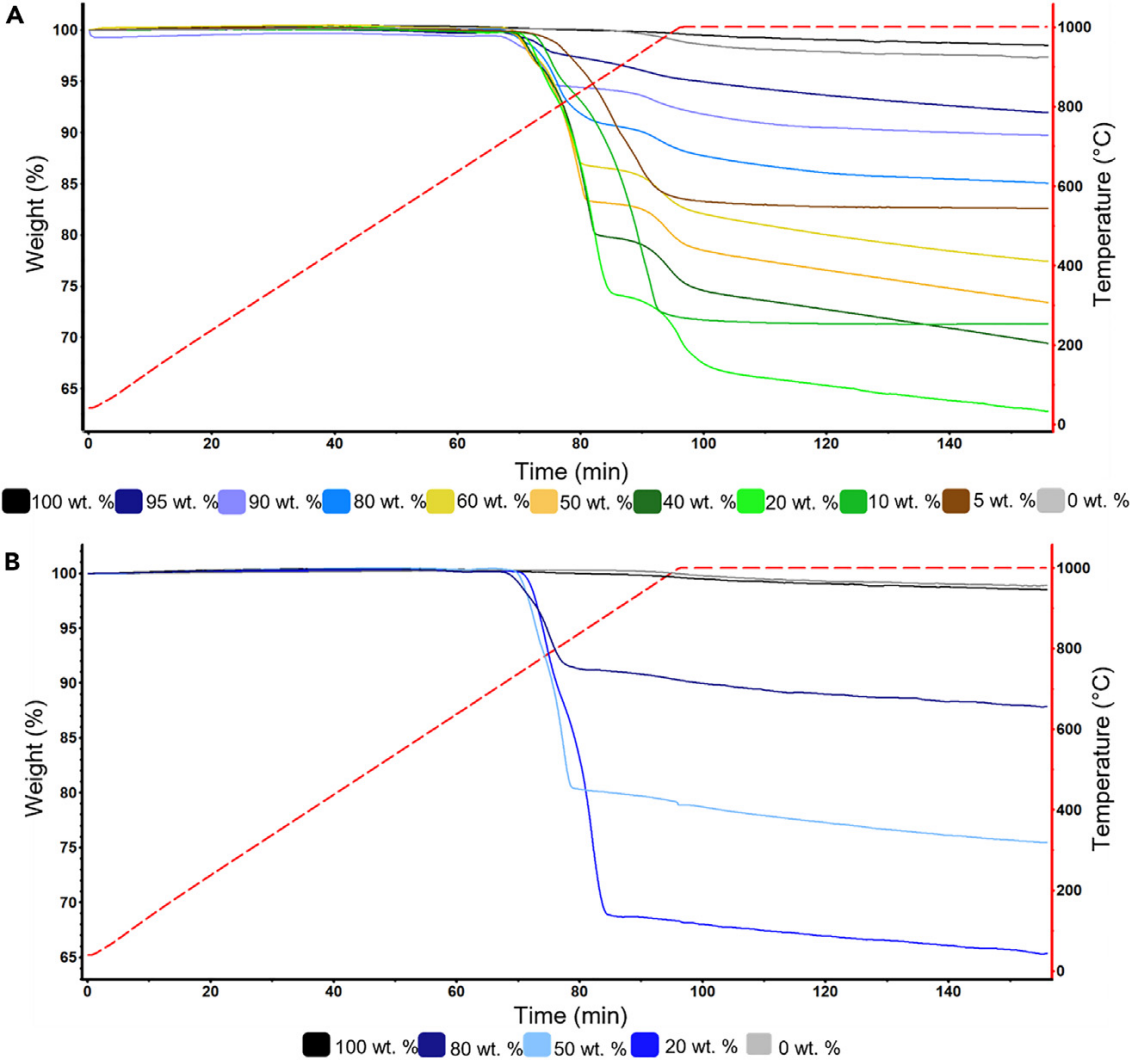
TGA of artificial black mass with different graphite compositions using (A) NMC and (B) LCO.

Once the reaction mechanisms of cathode and graphite were established, a series of TGA experiments were carried out on artificial BM samples with known composition. Figure 3A shows degradation curves for NMC at various graphite/cathode ratios. Pure graphite remains intact with only 1% mass loss, likely due to impurities. The same occurs for pure NMC: as no graphite is available to react with, only a mass loss of 2% at elevated temperature (>1000°C) was measured, which may be related to impurities. All degradation curves produced have a similar behavior to the one presented in Figure 1A. Invariably, reduction begins at 688°C ± 15°C, showing the highest mass loss at 773°C ± 38°C. Then a final step occurs that finishes at 1,000°C after 15 min of the start of the isothermal phase. Starting with the 95% graphite sample, the mass losses are minimal but clearly increasing as the concentration of NMC increases. This behavior is consistent until graphite concentration decreases down to 10 wt. %. At this concentration, the mass change is reduced, comparable to that at 20% graphite. For the artificial BM containing just 5% of graphite, there is also a smaller mass loss, comparable to the 80% graphite sample. This is a reasonable behavior of the system since the theoretical graphite/NMC ratio needed for the reduction reaction to occur is 0.11 (10 wt. % graphite). Mass loss increases as more O_2_ from the NMC is available to react with graphite. Nonetheless, once the molar ratio decreases under 0.11, the mass change percentage decreases, as graphite becomes the limiting reagent in the chemical reaction. This is an important phenomenon to identify since it sets the limits of graphite that can be estimated with this method.

The mass changes of LCO-standardized curves are analogous to those observed with NMC (Figure 3B). The reduction starts at 686°C ± 3°C. As in NMC, the mass loss increases as the concentration of graphite decreases, at least down to 20%. Although the temperatures at which mass loss was observed were the same, the thermoreduction rate was faster with LCO than in NMC. As mentioned earlier, the shape of LCO-containing artificial BM presents a single degradation step, unlike the various stages observed with NMC. This is an important outcome of this study since it shows that the distinction between TGA mass loss curves can also provide information about the dominant chemistry of the cathode particles.

**Figure 4:**
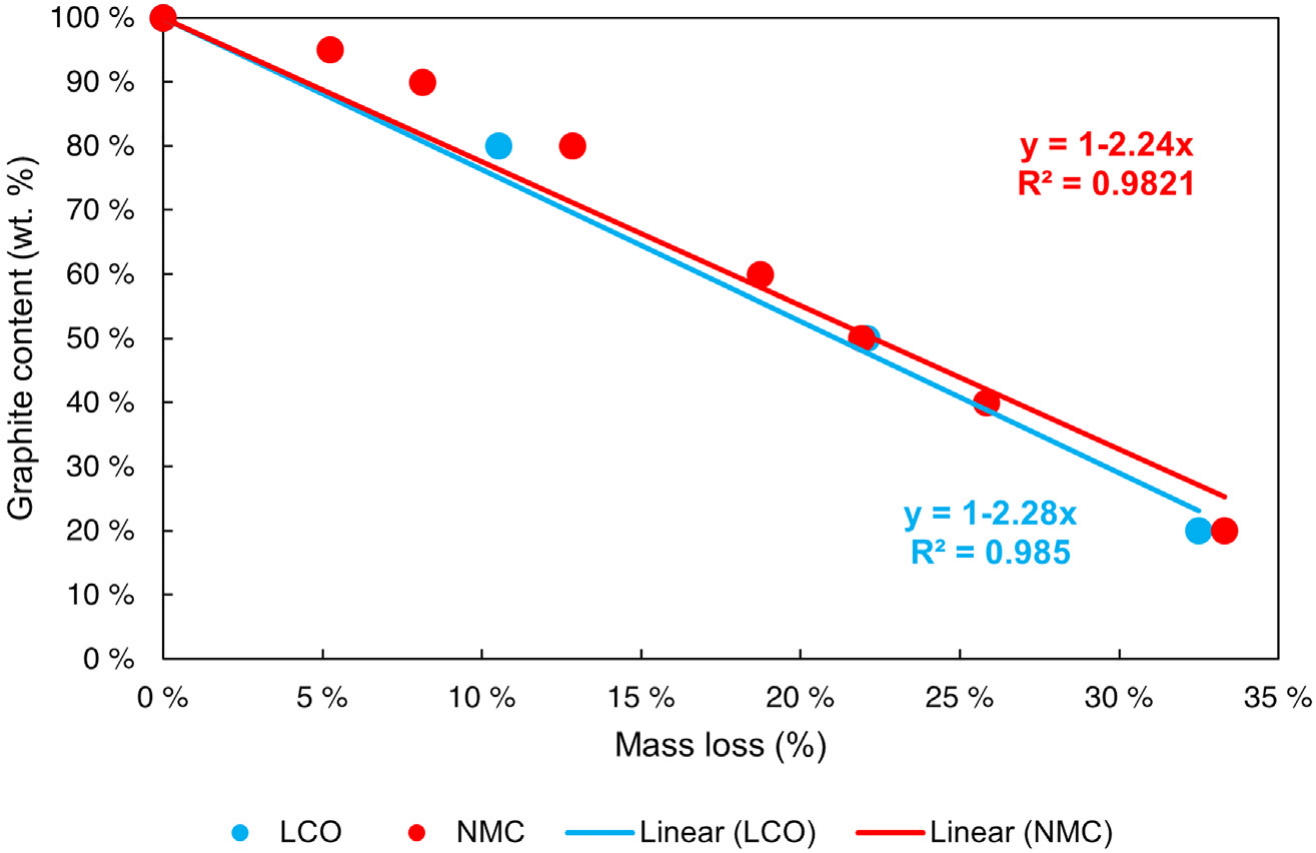
Graphite content linear correlation to mass loss in NMC and LCO artificial black mass, adjusted to 100% intercept.

The results with both artificial BMs show that, based on the chemical reduction of the cathode, the mass change can be correlated with the graphite content in the BM. To do this, only the mass change percentage starting from 685°C to the end of the experiment is considered, as this is the one associated with the chemical reaction of graphite. As a result, the graphite content can be expressed as a function of the mass change (Equation 6).

(Equation 6) $$y_{\alpha }=f\left(x_{\beta }\right)$$

where y_α_ is the graphite content in fraction basis and $x_{\beta }$ is the mass change measured by TGA also in fraction basis. Hence, when every mass change is evaluated in both cathode standard curves, a linear correlation is obtained as Figure 4 shows.

The exact mass loss for each graphite concentration obtained from the TGA can be consulted in Figures S2–S13 of the supplementary information. As seen in Figure 4, there is a linear correlation between the mass losses during thermal degradation and graphite content in both artificial BMs. Interestingly, the correlation obtained with both artificial BMs is similar within experimental error, suggesting that thermal degradation is more dependent on graphite content than on the cathode chemistry. The resulting Equations 7 and 8 can be used to estimate the graphite content based on the mass change that a sample suffers in a TGA under inert gas flow.

(Equation 7) $$y_{NMC}=1-2.24x_{NMC}$$

(Equation 8) $$y_{LCO}=1-2.28x_{LCO}$$

where "y” represents the fractional content of graphite and “x” represents the fractional mass change in the chemical reaction between the graphite and the cathode obtained from TGA.

### Characterization of industrial BM

**Figure 5:**
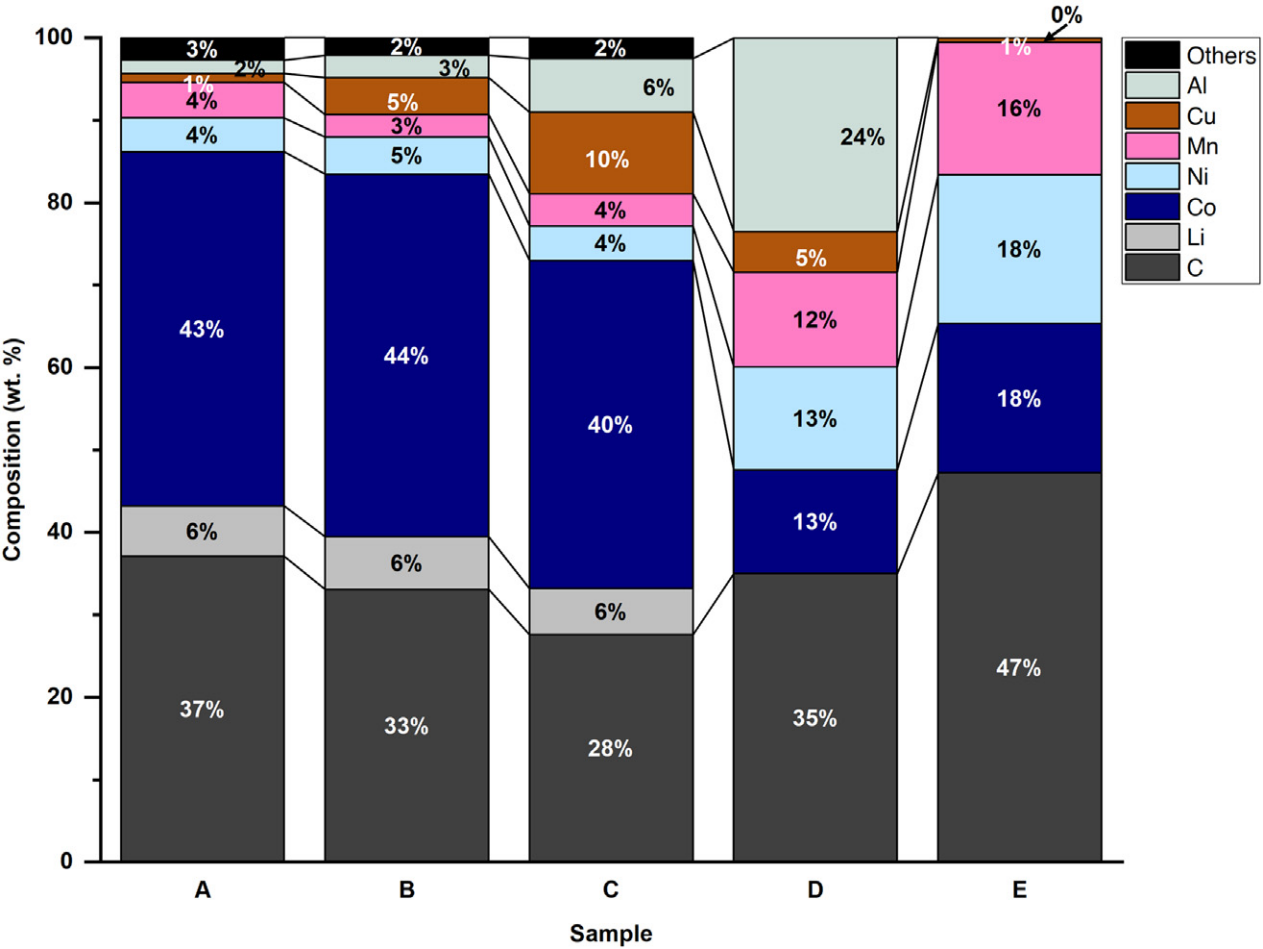
Elemental composition of Industrial BM samples.

To validate the methodology hereby proposed, five different samples of industrially produced BM were analyzed. Industrial BM likely deviates from the ideal behavior presented in the previous section, as it contains a myriad of impurities such as Cu, Al, polymers, and organic solvents.^13^^,^^14^ This likely affects the TGA-EGA characterization results since these impurities may promote additional chemical reactions. For example, Al acts as a reductant of most metal oxides in the battery since it has a strong tendency to oxidize, due to its low free Gibbs energy of formation. Indeed, Al is used as a reductant in industrial pyrometallurgical processes.^47^ The composition of the industrial BM studied in this work is presented in Figure 5.

**Figure 6:**
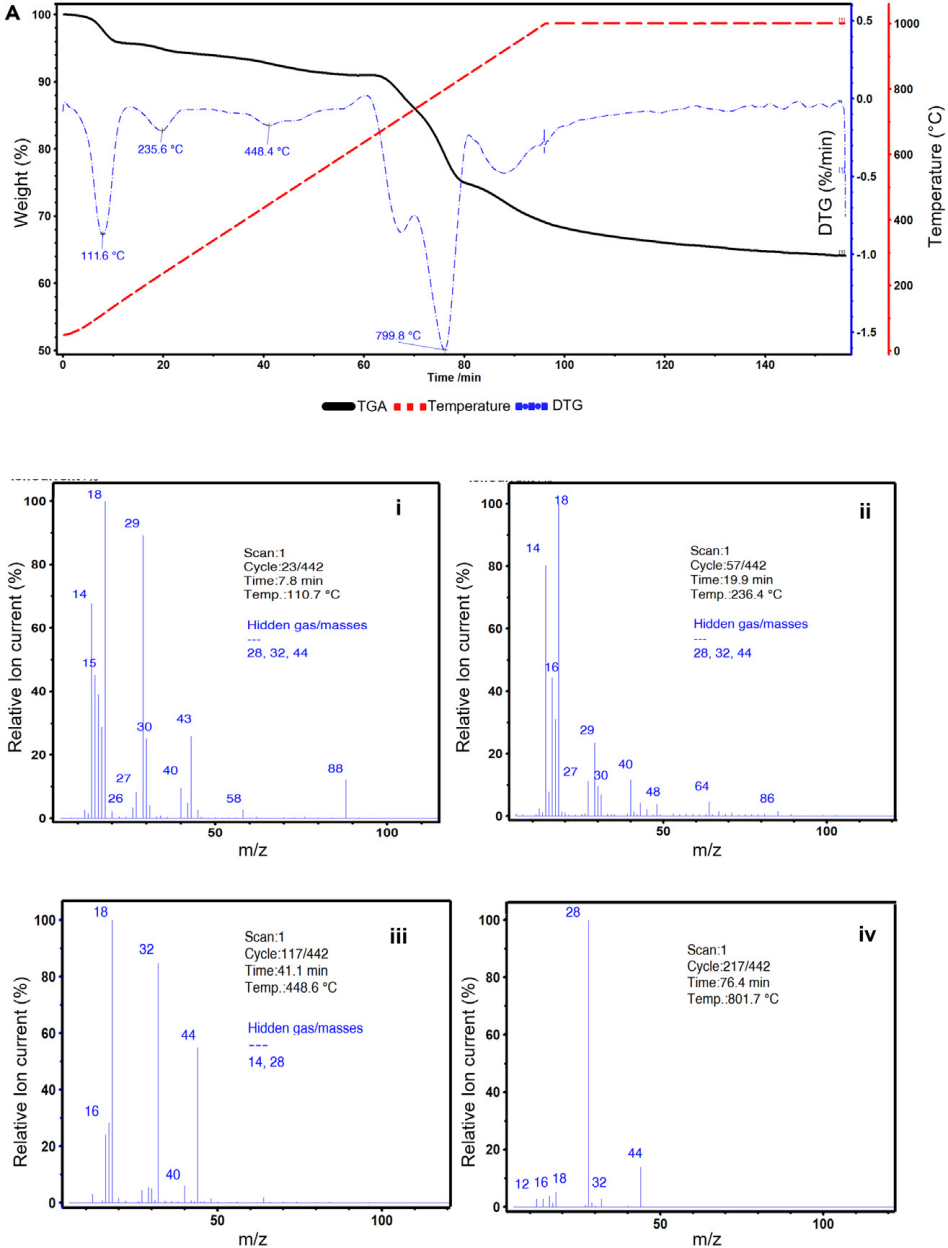
TGA-derivative thermogravimetry (DTG) of sample D industrial black mass (B), MS at first DTG peak (110.7°C) (i), at second DTG peak (236.4°C) (ii), at third DTG peak (448.6°C) (iii), and at fourth DTG peak (801.7°C) (iv).

All industrial BM samples were analyzed using TGA, and Sample D is presented to illustrate the results, as shown in Figure 6. The various degradation stages observed with this sample were also present in all other industrial BMs analyzed in this work (see supplementary information). The first derivative of the TGA was plotted to help identify the most relevant mass changes, showing maxima at 112°C, 236°C, 448°C, and 800°C. Mass spectroscopy (MS) was used to identify characteristic gas products evolving at each of these main degradation temperatures, as detailed in Table S1 of supplementary information. The two main gas components produced in the first stage of thermal decomposition (80°C–120°C) are water (m/z = 18) and ethylene carbonate (EC; m/z = 14, 29, 43, and 88), a solvent for the electrolyte of LIBs.^48^ This first stage resulted in a 4% mass loss. The presence of organic solvents indicates that the BM samples have not previously undergone thermal treatment.

The second stage of thermal degradation was centered at 236°C and resulted in a mass loss of ca. 2%. At this temperature, MS detected the production of water, traces of EC, other organic species such as diethyl carbonate (DEC), polycarbonate (PC) (See Table S1 in supplementary information for detailed information of m/z current ions), and phosphoryl fluoride (POF_3_) (m/z = 48, 64, and 86). POF_3_ is a known product of the chemical reaction between LiPF_6_ and H_2_O (Equation 9)^49^:

(Equation 9) $$LiPF_{6}+H_{2}O\;\to LiF+POF_{3}+2HF$$

The reaction products between LiPF_6_ and water at this temperature include LiF, POF_3_, and HF. From a safety perspective, POF_3_ and HF are of special interest since they are hazardous and corrosive.^49^ Thus, it is advised to consider these reactions whenever thermal processing of battery waste is planned.

In the following stage of mass loss, centered at 450°C, water evolution was still found, along with O_2_ (m/z = 16 and 32) and CO_2_ (m/z = 44). Comparatively weaker signals that correspond to trace amounts of polyvinylidene fluoride (PVDF) decomposition products and organic solvents such as dimethylformamide (DMF), dimethyl sulfoxide (DMSO), and N-methyl pyrrolidone (NMP) were identified. From industrial BM characterizations previously published by other authors, the total mass of active particles typically corresponds to 70%–90% of the total weight.^13^^,^^15^^,^^17^^,^^50^ This is confirmed by the results of this work where the impurities are estimated to be ca. 10% of the total sample mass.

**Figure 7:**
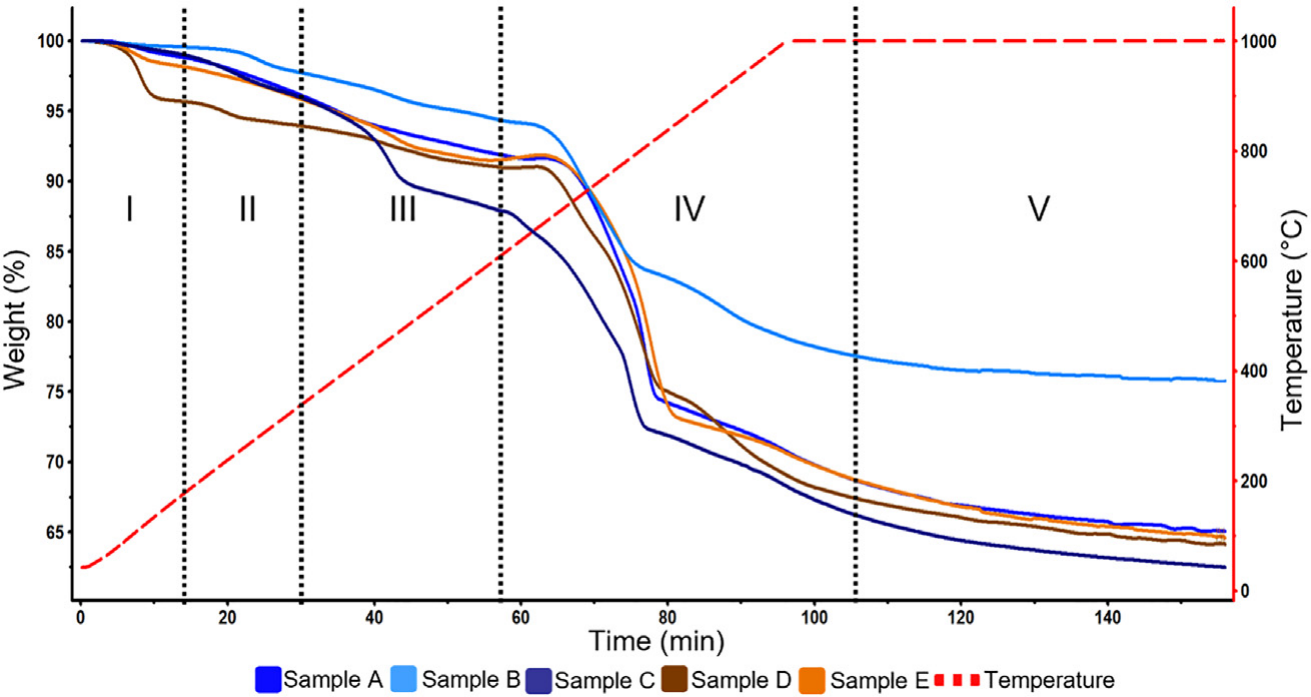
TGA of different industrial black mass samples.

At the last stage of degradation, a significant mass loss was observed, beginning at ca. 680°C and occurring rapidly in several distinct steps. This multiple-stage thermal degradation of graphite resembles the behavior observed in NMC-containing artificial BM discussed in the previous section. The equimolar composition of Co, Ni, and Mn from the elemental analysis presented in Figure 6 corroborates that NMC is likely the cathode chemistry in these samples. This corroborates that TGA can also help to identify the dominant cathode species in an unknown BM sample. At this temperature, the MS spectrum did not identify any more water, and the main species produced were CO (m/z = 28) and CO_2_ (m/z = 44). These signals coincide with those observed with the artificial BMs during the cathode reduction stage. As explained in the previous section, this is the stage used to estimate graphite content since the other mass losses correspond to thermal degradation of impurities. Finally, after the chemical reduction of cathode particles with graphite, the mass loss continues at a slower rate until reaching a plateau. It is possible that this corresponds to residual Mn that slowly reduces at high temperatures until the isothermal phase ends. As seen, there are differences between artificial BM and industrial BM samples due to the impurities found in the latter that need to be considered when quantifying graphite content from TGA results. Figure 7 compiles the TGA mass degradation profiles of the five different industrial BM samples. Each sample presents the same five distinctive stages, based on the identified in the analysis of sample D (shown in Figure 5). The exact detailed gas analysis, temperatures, and derivative thermogravimetry can be found in Table S1 of the supplementary information.

**Figure 8:**
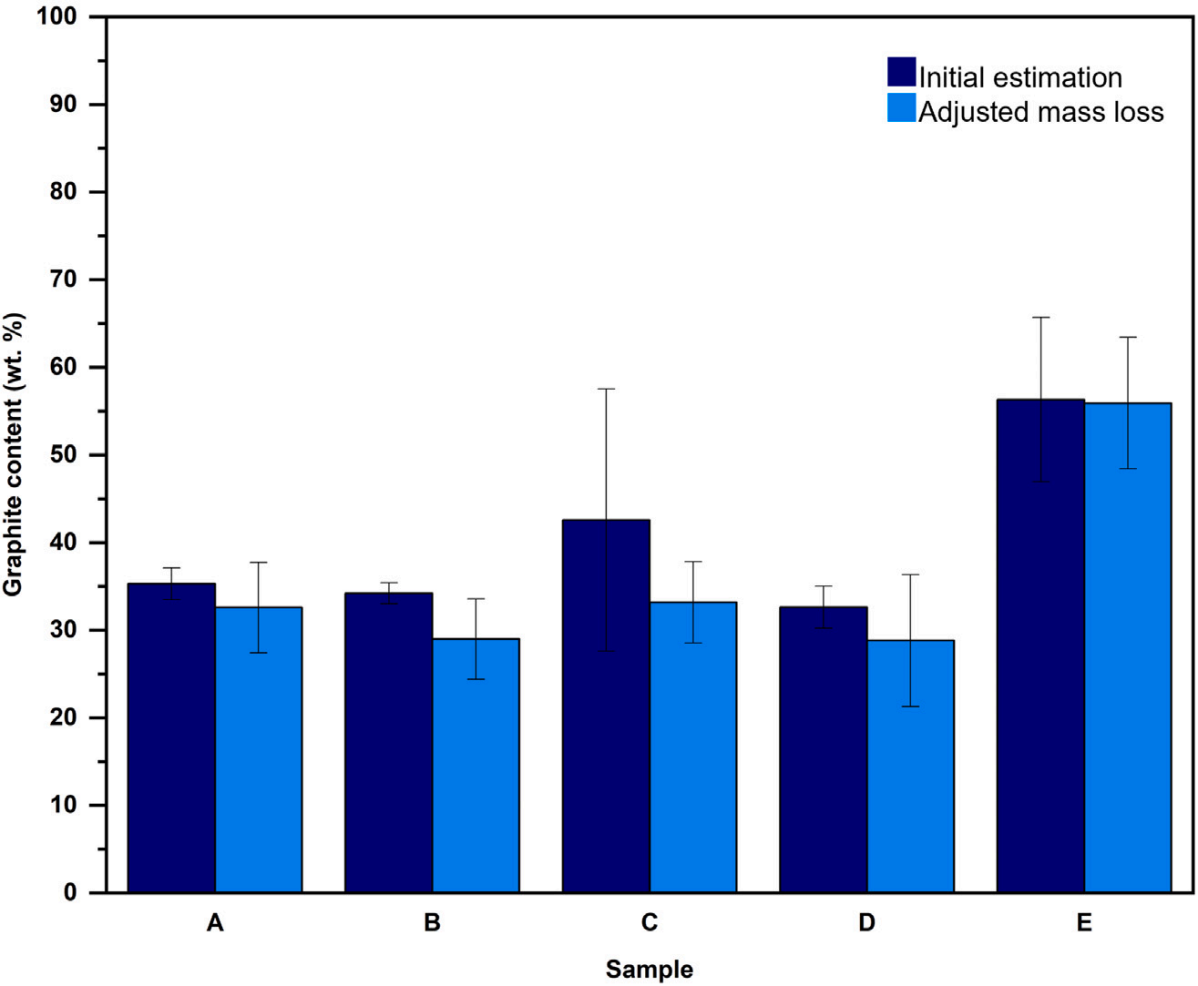
Graphite content calculated in industrial BM samples. Error bars represent the sensitivity error based on the graphite content measured by elemental analysis (shown in Figure 5).

Stage I invariably corresponds to the evaporation and decomposition of moisture, electrolytes, and other volatile organics such as DEC, dimethyl carbonate, and ethyl methyl carbonate. Stage II results from the decomposition of Li salt into H_3_PO_4_ and HF and from the thermal decomposition of polymers. Stage III corresponds to the decomposition of the binder in the cathode, along with polymer traces. Stage IV corresponds to the cathode reduction with graphite. The final Stage V is the isothermal phase, where mass loss is significantly small and occurs at a slow rate. For the estimation of graphite, only Stages IV and V should be considered, as these are the only regions of significance according to the analysis performed with artificial BM. Figure 8 shows the results of the estimation of the graphite content according to the method hereby proposed. Detailed values obtained from each sample are shown in Table S2 of supplementary information.

Following the reduction profiles shown in Figure 7, samples A, B, and C are considered to have mainly LCO cathode, an assumption further supported by their high Co content (Figure 5), although it is possible that other cathode chemistries are present in a lower proportion. In contrast, samples D and E exhibit three distinctive degradation profiles indicating an NMC cathode composition. Regarding the calculated graphite content in samples A and B, it is remarkably close with their measured carbon content. The first potential source of error is that the carbon analysis performed by CRS laboratories considered all carbon-containing species and not only graphitic carbon. In addition, metallic Cu was identified in all industrial BM samples. The presence of Cu in the industrial BM is significant because it represents an unreacted residual mass, thus affecting the relative mass loss attributed to the reaction between the cathode and graphite. This can introduce a significant source of error in samples with high concentration of pure metals such as samples C, D, and E. In an analogous manner, Fe, Zn, and P which are part of the “others” species in Figure 5 might influence the graphite estimation. Therefore, the mass losses were adjusted to consider the presence of impurities that influence the calculation, normalizing the mass content of active components by subtracting impurities from the total mass of the sample. For samples A and B, these corrections increase the error to 13%. However, for samples C, D, and E the corrected graphite content values are closer to the carbon content, and the estimated error is more consistent throughout all samples, representing a systematic error of the methodology. Nevertheless, further explanations are needed to understand the deviations in samples C and D. These samples report a significant content of Al, likely from the current collector foils in a battery cell. It is well known that Al is readily oxidized, generating species that are chemically stable even at elevated temperatures.^47^ For industrial BMs with a high Al content (e.g., Sample D) the graphite estimation may be affected since Al reacts competitively with graphite, but producing solid oxides that remain in the BM mixture. The presence of Al presents a significant challenge. One approach to mitigate the associated errors in future work is to generate new artificial BM mixtures, but with varying concentrations of elemental Al. This should enable the identification of mass changes resulting from the interaction between active particles and Al by comparing the curves of the active particles and graphite. At the same time, carbon black which is also present in cathode materials as a conductive additive in small quantities (10 wt. %)^51^ can also act as a reducing agent. As it has been shown in Fe oxides, carbon black has a strong reducing power, even better than graphite or other carbon materials.^52^ Furthermore, by introducing other elements like Cu, Fe, and organic compounds such as PVDF, the same effects can be observed on the curves. This study is a potential avenue for future research.

As seen, industrial BM graphite characterization is possible with TGA, provided that the impurities in the battery active materials mixture are properly accounted for. Unreactive metals such as Cu and competitive reducing agents such as Al influence the total mass losses. Nonetheless, the methodology presented here is simple and inexpensive, does not require any intensive sample preparation, and provides a quick estimation of graphite content with a low margin of error. Additionally, it can aid in identifying the dominant cathode chemistry in industrial BM that may impact subsequent separation and recovery strategies.

### Conclusion

A simple and inexpensive method to quantify graphite using TGA was developed by exploiting the reducing potential of graphite when mixed with LMOs. It was demonstrated that the thermal reduction reaction between the cathode and graphite at 650°C–1,000°C under inert atmosphere showed a mass loss linearly correlated with graphite content. NMC and LCO showed similar degradation profiles due to their comparable reducible metal content. Standardized curves for artificial BM containing NMC and LCO produced two consistent equations to quantify graphite content. The method developed is limited to a minimum of 20% graphite composition to prevent it from becoming the limiting reagent. Admittedly, some accuracy of the method is lost with industrial BM due to the presence of cathode chemistries and other components. To quantify graphite in industrial BM, we suggest the following methodology.

1. Conduct TGA on the sample under the conditions described in the STAR methods section.
2. Compare the resulting mass change curve with standardized curves for LCO and NMC cathode chemistries (Figure 3) to determine the type of chemistry present.
3. Account for the mass losses due to volatile and organic impurities up to 650°C.
4. Estimate the relative mass degradation in the high-temperature region corresponding to the reduction of cathode materials and the evolution of CO and CO_2_, up to 1,000°C
5. Calculate the fraction of graphite present in the sample using either Equations 7 or 8 depending on the type of cathode.

Although there are limitations to the minimum quantity of graphite that can be accurately quantified using this method, the consistency of the observed error suggests that it can be further studied and potentially improved. Future research may focus on the effect of impurities, such as carbon black, AI, Cu, or other metals, optimizing the methodology to increase the sensitivity and reduce the minimum detectable quantity of graphite, while also identifying factors that may influence the accuracy and precision of the measurements. Additionally, since other cathode materials, such as LFP, LMO, and lithium titanate, may exhibit different reduction behaviors, new standardized curves must be defined to ensure accurate graphite estimation. Nonetheless, the proposed method still holds promise as a fast and simple approach for estimating graphite content with samples with more than 20 wt. % graphite and for comparing relative changes in graphite content between different battery waste samples.

### Limitations of the study

The methodology presented in this study offers a quick and straightforward approach to characterize graphite using TGA in industrial BM. Nonetheless, it is important to acknowledge that the influence of impurities such as conductive additives, current collectors, plastics, and other components requires further investigation to reduce potential error and increase the overall accuracy.

## STAR★Methods

### Key resources table

**Table:** 

REAGENT or RESOURCE	SOURCE	IDENTIFIER
**Chemicals, peptides, and recombinant proteins**		

Li(Ni_0.33_Mn_0.33_Co_0.33_)O_2_ (NMC)	MSE Supplies	CAS: 346417-97-8
LiCoO_2_ (LCO)	MSE Supplies	CAS: 12190-79-3
Spherical graphite	Prographite	CAS: 7782-42-5
He (99.99%)	Linde	CAS: 7440-59-7
N_2_ (99.99%)	Linde	CAS: 7727-37-9

**Software and algorithms**		

Origin 2023	OriginLab	https://originlab.com
Proteus Analysis	NETZSCH	https://analyzing-testing.netzsch.com/en/products/software/proteus

### Resource availability

#### Lead contact

Further information should be directed to and will be fulfilled by the lead contact, Rodrigo Serna (rodrigo.serna@aalto.fi).

#### Materials availability

This study did not generate new unique reagents.

### Method details

#### Materials

Two sets of artificial black mass samples were prepared by weighting 5 g of each sample in a Radwag PS1000R scale according to the following ratios. The first set was prepared with compositions of 0, 5, 10, 20, 40, 50, 60, 80, 90, 95 and 100 wt. % NMC, the rest being graphite. The second set was prepared with compositions of 0, 20, 50, 80 and 100 wt. % LCO mixed with graphite. The reagents were manually mixed in a plastic container using a metal spatula until visual homogeneity was achieved. Subsequently, manual agitation was performed for 3 min. Industrial black mass samples (“A”, “B”, “C”, “D”, and “E”) were obtained from a local battery recycling facility with unknown cathode chemistries and without specification regarding pre-processing conditions. The compositional analysis of “A, “B”, and “C” black mass samples were commissioned to an external laboratory. CRS Laboratories conducted elemental analysis via four acid digestion, multielement analysis with ICP-OES, and combustion analysis for TC using a Leco analyzer. Samples D and E were analyzed with portable X-ray fluorescence machine (Oxford Instruments, X-MET 5000).

#### Thermogravimetric analysis

TGA-EGA was conducted in a Netzsch STA 449 coupled with a quadrupole mass spectrometer (QMS 403 Aëolos Quadro) for evolved gas analysis. Samples of 25 ± 5 mg were loaded on an alumina crucible under a He atmosphere (99.99%, Linde) with a 70 mL/min flow. It is important to remark that He needs to be used as an inert atmosphere. In preliminary tests using N_2_ gas (not presented here), MS ion signals in m/z = 28 and 14 were detected, which overlap with those of CO (m/z = 28), an important molecule to monitor.^37^ The TGA was programmed to heat the sample at a rate of 10 °C/min up to 1000°C followed by isothermal stabilization for 60 min. It is worth mentioning that at temperatures >1100°C, a reaction between the cathode particles and the alumina crucible occurs and it is recommended to avoid exceeding 1000°C (See Figure S1 in supplemental information).

#### Morphological characterization

Thermal treatment experiments with a larger mass than that of TGA were performed in a Tube Furnace (Lenton LFT15/450) and the materials were characterized using a Tescan Mira 3 scanning electron microscope with an Oxford energy dispersive X-ray Spectroscopy (EDS) detector. For this analysis, 1 g sample of artificial BM containing a 1:1 mixture of NMC and graphite was used. In this case, N_2_ was used as an inert atmosphere under a heating rate of 5°C/min. While the thermal treatment conditions in this characterization differed from those used in TGA, the use of N_2_ as an inert atmosphere and a slower heating rate are unlikely to significantly impact the morphological characterization results. Furthermore, the same dynamic and isothermal phases were conducted on the sample, i.e., heating up to 1000°C followed by a thermal stabilization phase for 60 min. To facilitate mounting, a carbon-coated tape was placed on an SEM stub, onto which the sample powder was manually adhered. Any excess material was then removed using compressed air. The samples were not sputter-coated as the sample powder contains graphite that is naturally conductive. The carbon-coated tape was only used for facilitating the mounting process, allowing the sample powder to be adhered to the SEM stub efficiently. The SEM stub, now containing the powder sample, was subsequently loaded for analysis. For imaging purposes, the SEM imaging parameters were configured with an accelerating voltage of 15 kV and two distinct magnifications (1.2 kX and 3.5 kX), utilizing the secondary electron detector (SED) and back-scattered electron (BSE) for Figures 2A–2D respectively.

## Data Availability

• Data reported in this paper will be shared by the lead contact upon request. • This paper does not report original code. • Any additional information required to reanalyze the data reported in this paper is available from the lead contact upon request.

## References

[1] Carrara S., Alves Dias P., Plazzotta B., Pavel C. (2020). Raw Materials Demand for Wind and Solar PV Technologies in the Transition Towards a Decarbonised Energy system. EUR 30095 EN.

[2] Jones B., Elliott R.J.R., Nguyen-Tien V. (2020). The EV revolution: The road ahead for critical raw materials demand. Appl. Energy.

[3] Buchholz P., Brandenburg T. (2018). Demand, Supply, and Price Trends for Mineral Raw Materials Relevant to the Renewable Energy Transition Wind Energy, Solar Photovoltaic Energy, and Energy Storage. Chem. Ing. Tech..

[4] Hameer S., van Niekerk J.L. (2015). A review of large-scale electrical energy storage. Int. J. Energy Res..

[5] Chen T., Jin Y., Lv H., Yang A., Liu M., Chen B., Xie Y., Chen Q. (2020). Applications of Lithium-Ion Batteries in Grid-Scale Energy Storage Systems. Trans. Tianjin Univ..

[6] Xu C., Dai Q., Gaines L., Hu M., Tukker A., Steubing B. (2020). Future material demand for automotive lithium-based batteries. Commun. Mater..

[7] Communication from the Commission to the European Parliament, the Council, the European Economic and Social Committee and the Committee of the Regions: Critical Raw Materials Resilience - Charting a Path towards Greater Security and Sustainability. (2020).

[8] Velázquez-Martínez O., Valio J., Santasalo-Aarnio A., Reuter M., Serna-Guerrero R. (2019). A Critical Review of Lithium-Ion Battery Recycling Processes from a Circular Economy Perspective. Batteries.

[9] Manthiram A. (2020). A reflection on lithium-ion battery cathode chemistry. Nat. Commun..

[10] Sommerville R., Shaw-Stewart J., Goodship V., Rowson N., Kendrick E. (2020). A review of physical processes used in the safe recycling of lithium ion batteries. Sustain. Mater. Technol..

[11] Gaines L. (2014). The future of automotive lithium-ion battery recycling: Charting a sustainable course. Sustain. Mater. Technol..

[12] Kaya M. (2022). State-of-the-art lithium-ion battery recycling technologies. Circ. Econ..

[13] Vanderbruggen A., Gugala E., Blannin R., Bachmann K., Serna-Guerrero R., Rudolph M. (2021). Automated mineralogy as a novel approach for the compositional and textural characterization of spent lithium-ion batteries. Miner. Eng..

[14] Vassura I., Morselli L., Bernardi E., Passarini F. (2009). Chemical characterisation of spent rechargeable batteries. Waste Manag..

[15] Ruismäki R., Rinne T., Dańczak A., Taskinen P., Serna-Guerrero R., Jokilaakso A. (2020). Integrating Flotation and Pyrometallurgy for Recovering Graphite and Valuable Metals from Battery Scrap. Metals.

[16] Salces A.M., Bremerstein I., Rudolph M., Vanderbruggen A. (2022). Joint recovery of graphite and lithium metal oxides from spent lithium-ion batteries using froth flotation and investigation on process water re-use. Miner. Eng..

[17] Vanderbruggen A., Sygusch J., Rudolph M., Serna-Guerrero R. (2021). A contribution to understanding the flotation behavior of lithium metal oxides and spheroidized graphite for lithium-ion battery recycling. Colloids Surf. A Physicochem. Eng. Asp..

[18] Liu J., Wang H., Hu T., Bai X., Wang S., Xie W., Hao J., He Y. (2020). Recovery of LiCoO2 and graphite from spent lithium-ion batteries by cryogenic grinding and froth flotation. Miner. Eng..

[19] Zhan R., Oldenburg Z., Pan L. (2018). Recovery of active cathode materials from lithium-ion batteries using froth flotation. Sustain. Mater. Technol..

[20] Qiu H., Peschel C., Winter M., Nowak S., Köthe J., Goldmann D. (2022). Recovery of Graphite and Cathode Active Materials from Spent Lithium-Ion Batteries by Applying Two Pretreatment Methods and Flotation Combined with a Rapid Analysis Technique. Metals.

[21] Liu J., Shi H., Hu X., Geng Y., Yang L., Shao P., Luo X. (2022). Critical strategies for recycling process of graphite from spent lithium-ion batteries: A review. Sci. Total Environ..

[22] (2004). Infrared Spectroscopy: Fundamentals and Applications.

[23] Harris D.C. (2010).

[24] XPS Instrumentation (2011).

[25] Bottom R. (2008). Principles and Applications of Thermal Analysis.

[26] (2011). Standard test methods for determination of carbon, sulfur, nitrogen, and oxygen in steel, iron, nickel, and cobalt alloys by various combustion and fusion techniques. https://www.astm.org/e1019-08.html.

[27] Pavia D.L., Lampman G.M., Kriz G.S., Vyvyan J.A. (2008).

[28] Ribeiro T., Brandao P. (2017). Development and validation of graphitic carbon analysis of graphite ore samples. Tecnol. Em Metal. Mater. E Min..

[29] Zhang Z., Schniepp H.C., Adamson D.H. (2019). Characterization of graphene oxide: Variations in reported approaches. Carbon.

[30] Harris P.J.F. (2018). Transmission Electron Microscopy of Carbon: A Brief History. Chimia.

[31] Mundszinger M., Farsi S., Rapp M., Golla-Schindler U., Kaiser U., Wachtler M. (2017). Morphology and texture of spheroidized natural and synthetic graphites. Carbon.

[32] Rinne T., Klemettinen A., Klemettinen L., Ruismäki R., O’Brien H., Jokilaakso A., Serna-Guerrero R. (2022). Recovering Value from End-of-Life Batteries by Integrating Froth Flotation and Pyrometallurgical Copper-Slag Cleaning. Metals.

[33] Peng C., Hamuyuni J., Wilson B.P., Lundström M. (2018). Selective reductive leaching of cobalt and lithium from industrially crushed waste Li-ion batteries in sulfuric acid system. Waste Manag..

[34] Mousa E., Hu X., Ånnhagen L., Ye G., Cornelio A., Fahimi A., Bontempi E., Frontera P., Badenhorst C., Santos A.C. (2023). Characterization and Thermal Treatment of the Black Mass from Spent Lithium-Ion Batteries. Sustainability.

[35] Zhan R., Pan L. (2022). A cycling-insensitive recycling method for producing lithium transition metal oxide from Li-ion batteries using centrifugal gravity separation. Sustain. Mater. Technol..

[36] Hanisch C., Loellhoeffel T., Diekmann J., Markley K.J., Haselrieder W., Kwade A. (2015). Recycling of lithium-ion batteries: a novel method to separate coating and foil of electrodes. J. Clean. Prod..

[37] National Institute of Standards and Technology. Chemistry WebBook. https://webbook.nist.gov.

[38] Babanejad S., Ahmed H., Andersson C., Samuelsson C., Lennartsson A., Hall B., Arnerlöf L. (2022). High-Temperature Behavior of Spent Li-Ion Battery Black Mass in Inert Atmosphere. J. Sustain. Metall..

[39] Chase M.W. (1998).

[40] (2023). NMC | 346417-97-8 ChemicalBook. https://www.chemicalbook.com/ChemicalProductProperty_EN_CB02750886.htm.

[41] Peng C., Liu F., Wang Z., Wilson B.P., Lundström M. (2019). Selective extraction of lithium (Li) and preparation of battery grade lithium carbonate (Li2CO3) from spent Li-ion batteries in nitrate system. J. Power Sources.

[42] Li J., Wang G., Xu Z. (2016). Environmentally-friendly oxygen-free roasting/wet magnetic separation technology for in situ recycling cobalt, lithium carbonate and graphite from spent LiCoO2/graphite lithium batteries. J. Hazard Mater..

[43] Lei S., Zhang Y., Song S., Xu R., Sun W., Xu S., Yang Y. (2021). Strengthening Valuable Metal Recovery from Spent Lithium-Ion Batteries by Environmentally Friendly Reductive Thermal Treatment and Electrochemical Leaching. ACS Sustain. Chem. Eng..

[44] Yue Y., Wei S., Yongjie B., Chenyang Z., Shaole S., Yuehua H. (2018). Recovering Valuable Metals from Spent Lithium Ion Battery via a Combination of Reduction Thermal Treatment and Facile Acid Leaching. ACS Sustain. Chem. Eng..

[45] González Y.C., Barrios O.C., González J.A., Barbosa L.I. (2022). Study on the carboreduction of the cathode material present in spent LIBs to produce Li2CO3 and CoO. Miner. Eng..

[46] (2017). 12190-79-3 CAS MSDS (LITHIUM COBALT(III) OXIDE) melting point boiling point density CAS chemical properties. https://www.chemicalbook.com/ChemicalProductProperty_US_CB7425354.aspx.

[47] Fichte R. (2000). Ullmann’s Encyclopedia of Industrial Chemistry.

[48] Xu K. (2004). Nonaqueous Liquid Electrolytes for Lithium-Based Rechargeable Batteries. Chem. Rev..

[49] Cavell R.G. (1968). Chemistry of phosphorus fluorides. Part III. The reaction of thiophosphoryl-fluoride with dimethylamine and some properties of the dimethylaminothio- phosphoryl fluorides. Can. J. Chem..

[50] Vanderbruggen A., Hayagan N., Bachmann K., Ferreira A., Werner D., Horn D., Peuker U., Serna-Guerrero R., Rudolph M. (2022). Lithium-Ion Battery Recycling–;Influence of Recycling Processes on Component Liberation and Flotation Separation Efficiency. ACS EST Eng.

[51] Qi X., Blizanac B., DuPasquier A., Lal A., Niehoff P., Placke T., Oljaca M., Li J., Winter M. (2014). Influence of Thermal Treated Carbon Black Conductive Additive on the Performance of High Voltage Spinel Cr-Doped LiNi0.5Mn1.5O4 Composite Cathode Electrode. J. Electrochem. Soc..

[52] Molaei M.J., Ataie A., Raygan S., Picken S.J. (2018). The effect of different carbon reducing agents in synthesizing barium ferrite/magnetite nanocomposites. Mater. Chem. Phys..

