# Supplementary material for: A simple methodology for the quantification of graphite in end-of-life lithium-ion batteries using thermogravimetric analysis
Source: iScience. 2023 Aug 29;26(10):107782. doi: 10.1016/j.isci.2023.107782 (PMC10507233; doi:10.1016/j.isci.2023.107782)
Supplement: Document S1. Figures S1–S14 and Tables S1 and S2 [file mmc1.pdf]

iScience, Volume *26*

Supplemental information

A simple methodology for the quantification of graphite in end-of-life lithium-ion batteries using thermogravimetric analysis

Luis Arturo Gomez-Moreno, Anna Klemettinen, and Rodrigo Serna-Guerrero

Supplemental information

A simple methodology for the quantification of graphite in end-of-life lithium-ion batteries using thermo-gravimetric analysis

Luis Arturo Gomez-Moreno, Anna Klemettinen, Rodrigo Serna-Guerrero

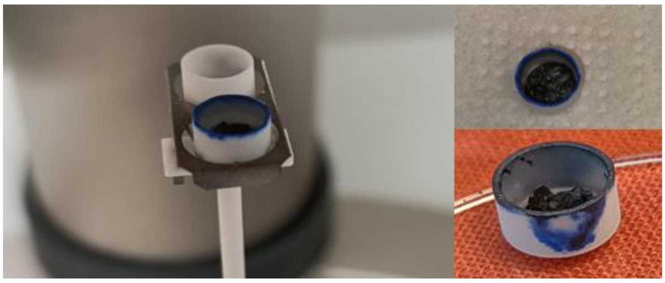

Figure S1. Reaction between the alumina crucible and NMC/Graphite black mass at elevated temperatures (> 1100°C) under He atmosphere, related to Materials and Methods section.

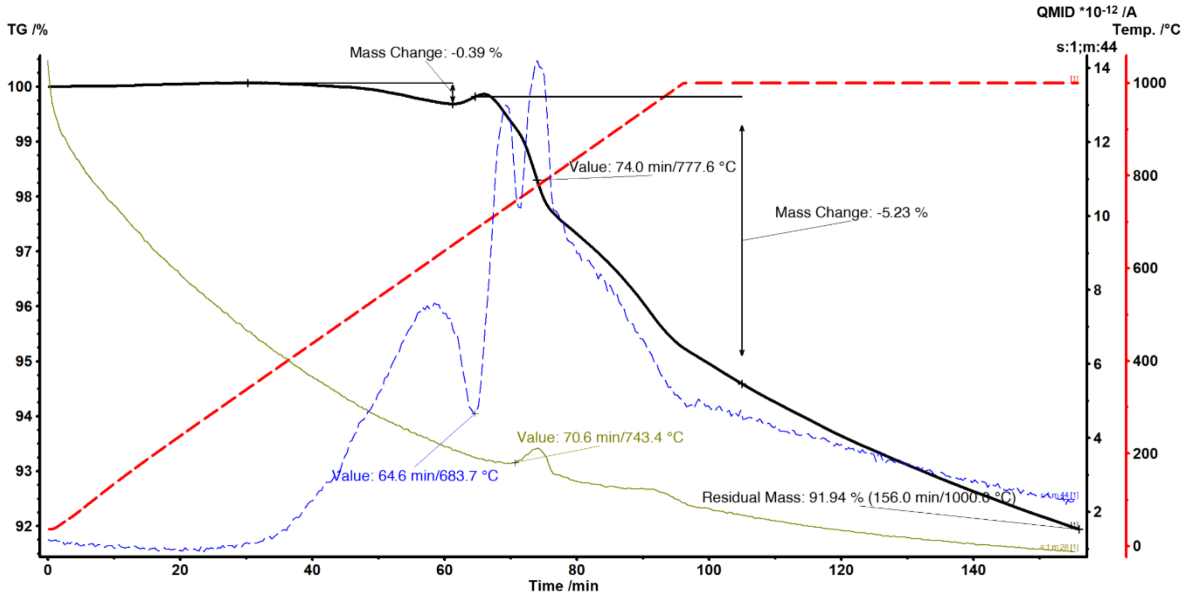

Figure S2. TGA-MS of NMC model black mass (95 wt. % graphite) and MS ion current curve of CO, and CO_2, related to Figure 4.

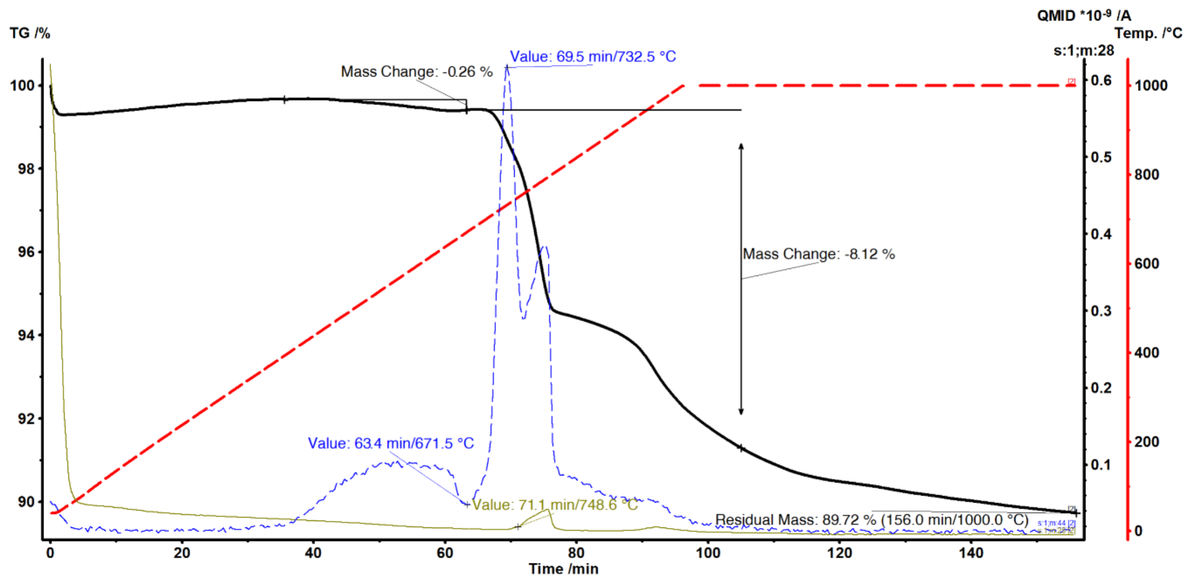

Figure S3. TGA-MS of NMC model black mass (90 wt. % graphite) and MS ion current curve of CO, and CO_2, related to Figure 4.

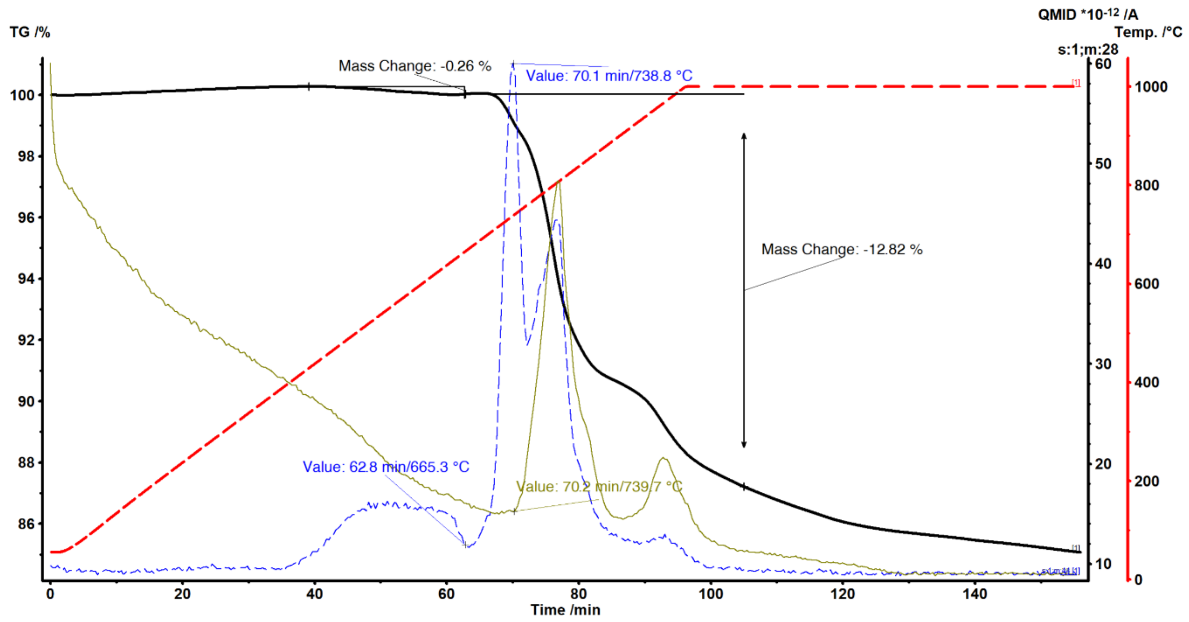

Figure S4. TGA-MS of NMC model black mass (80 wt. % graphite) and MS ion current curve of CO, and CO_2, related to Figure 4.

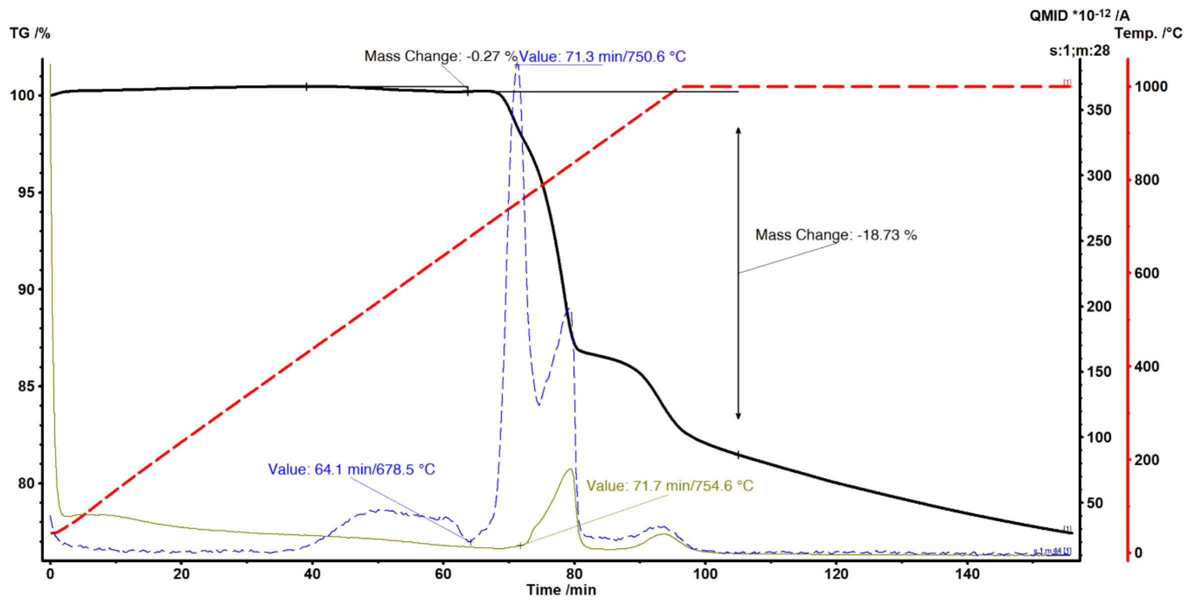

Figure S5. TGA-MS of NMC model black mass (60 wt. % graphite) and MS ion current curve of CO, and CO_2, related to Figure 4.

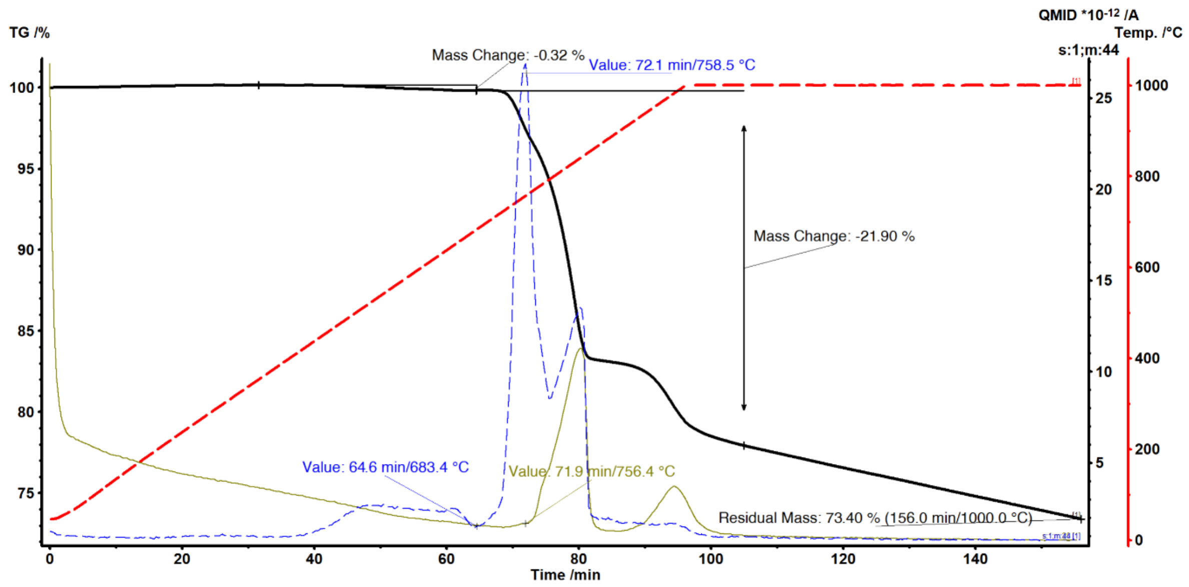

Figure S6. TGA-MS of NMC model black mass (50 wt. % graphite) and MS ion current curve of CO, and CO_2, related to Figure 4.

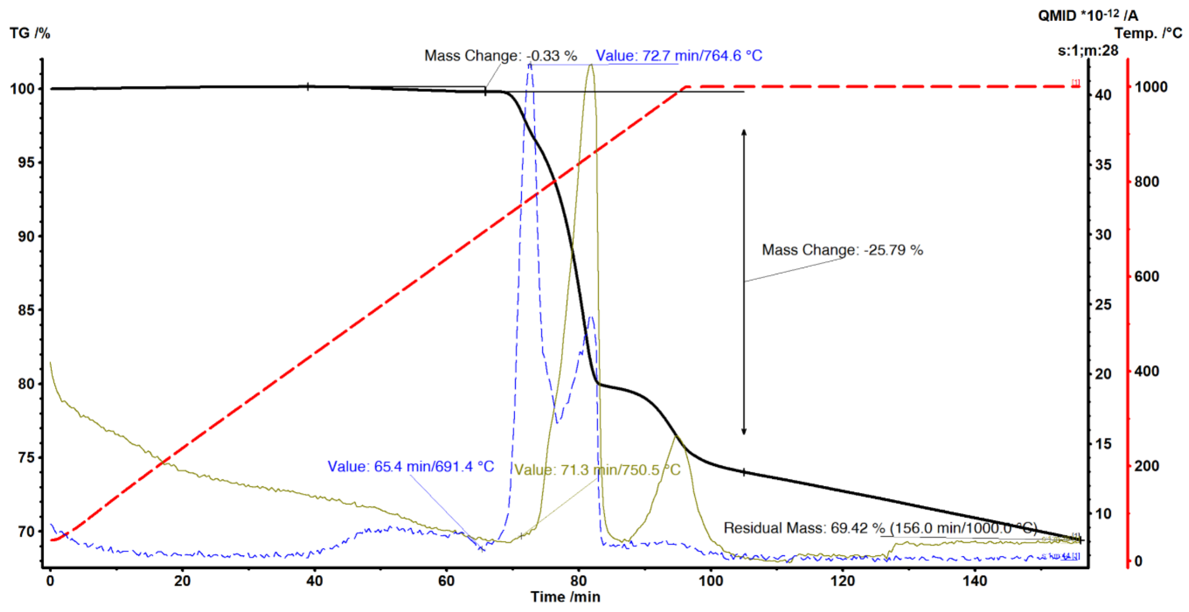

Figure S7. TGA-MS of NMC model black mass (40 wt. % graphite) and MS ion current curve of CO, and CO_2, related to Figure 4.

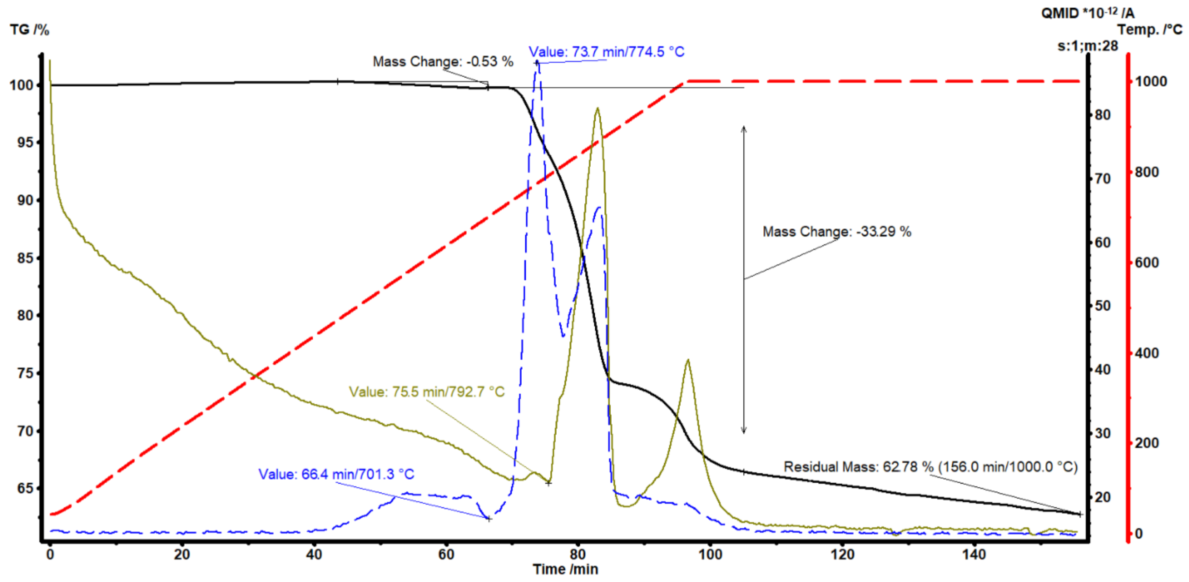

Figure S8. TGA-MS of NMC model black mass (20 wt. % graphite) and MS ion current curve of CO, and CO_2, related to Figure 4.

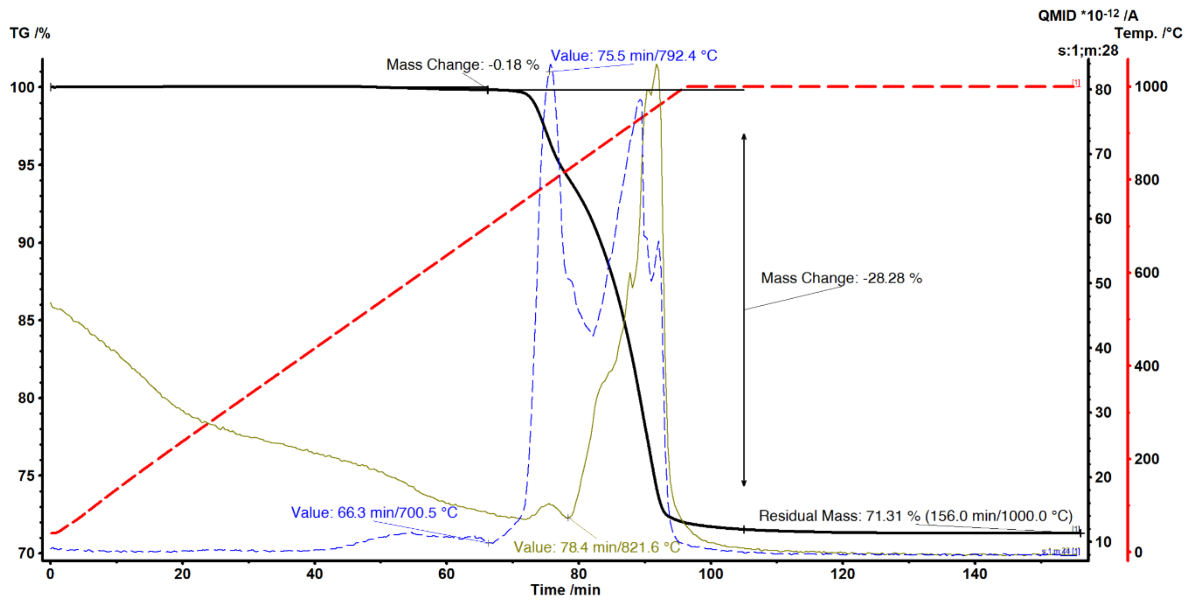

Figure S9. TGA-MS of NMC model black mass (10 wt. % graphite) and MS ion current curve of CO, and CO_2, related to Figure 4.

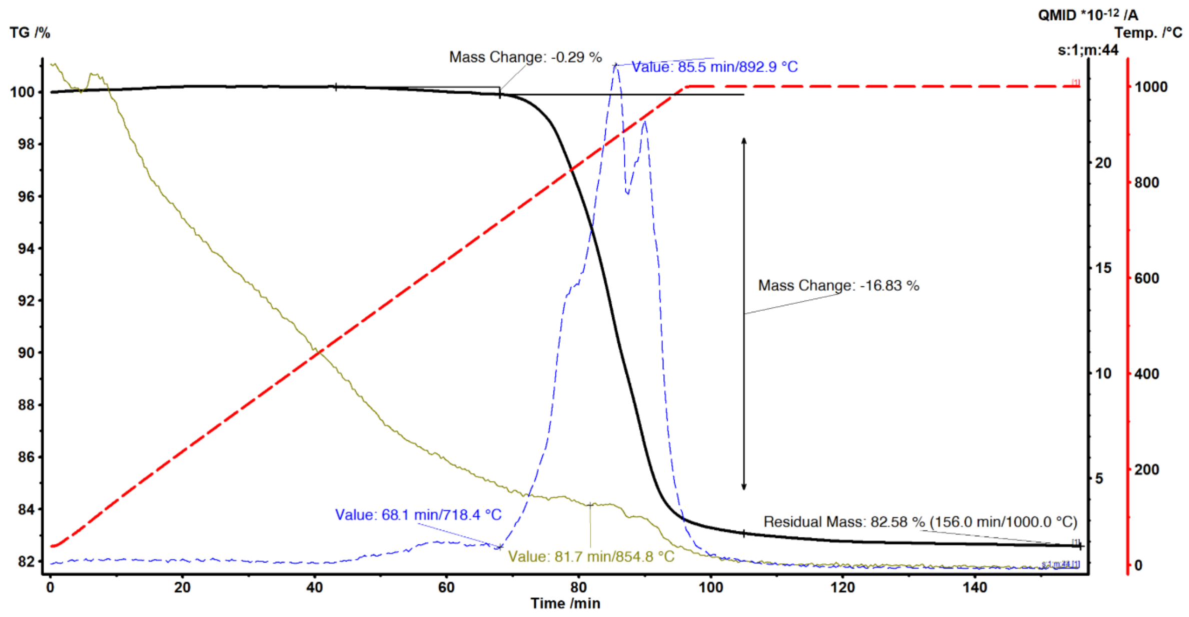

Figure S10. TGA-MS of NMC model black mass (5 wt. % graphite) and MS ion current curve of CO (28), and CO_2 (44), related to Figure 4.

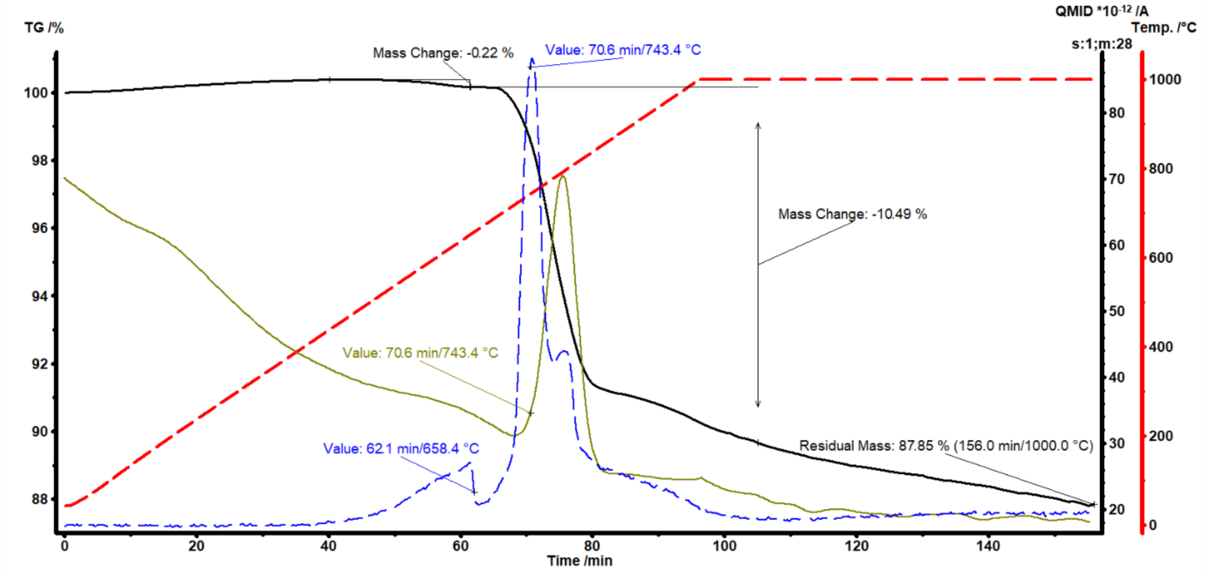

Figure S11. TGA-MS of LCO model black mass (80 wt. % graphite) and MS ion current curve of CO (28) and CO_2 (44), related to Figure 4.

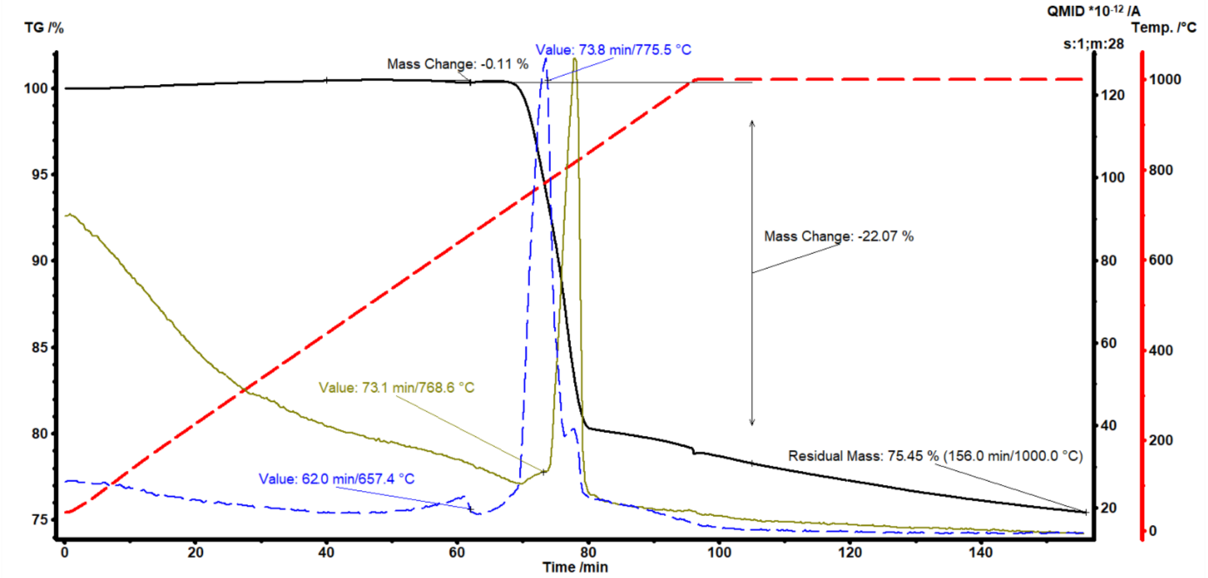

Figure S12. TGA-MS of LCO model black mass (50 wt. % graphite) and MS ion current curve of CO (28) and CO_2 (44), relate to Figure 4.

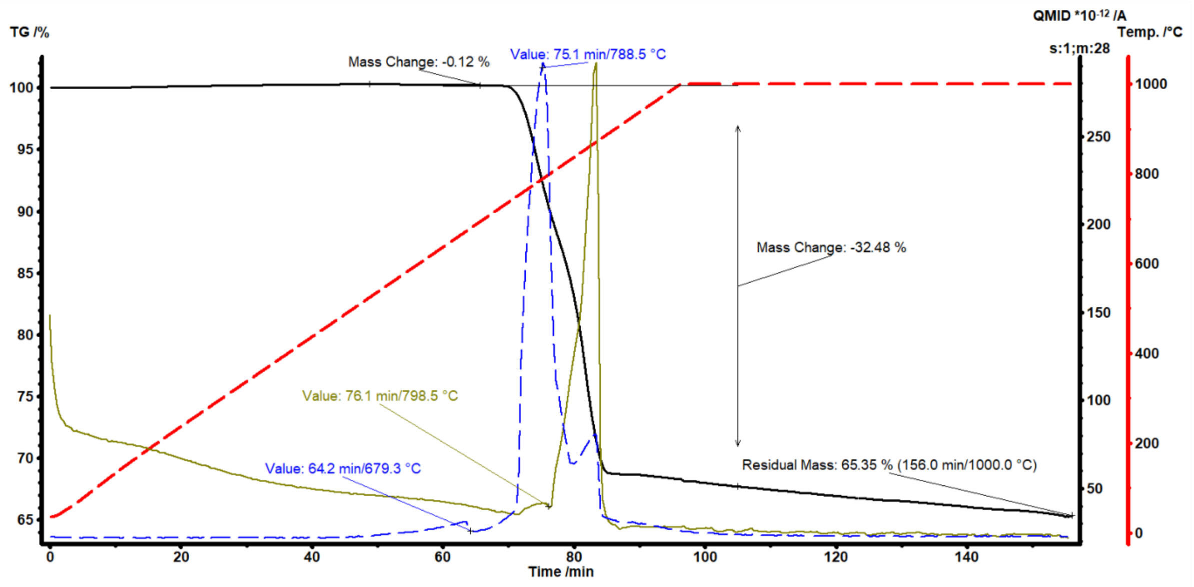

Figure S13. TGA-MS of LCO model black mass (20 wt. % graphite) and MS ion current curve of CO (28) and CO_2 (44), relate to Figure 4.

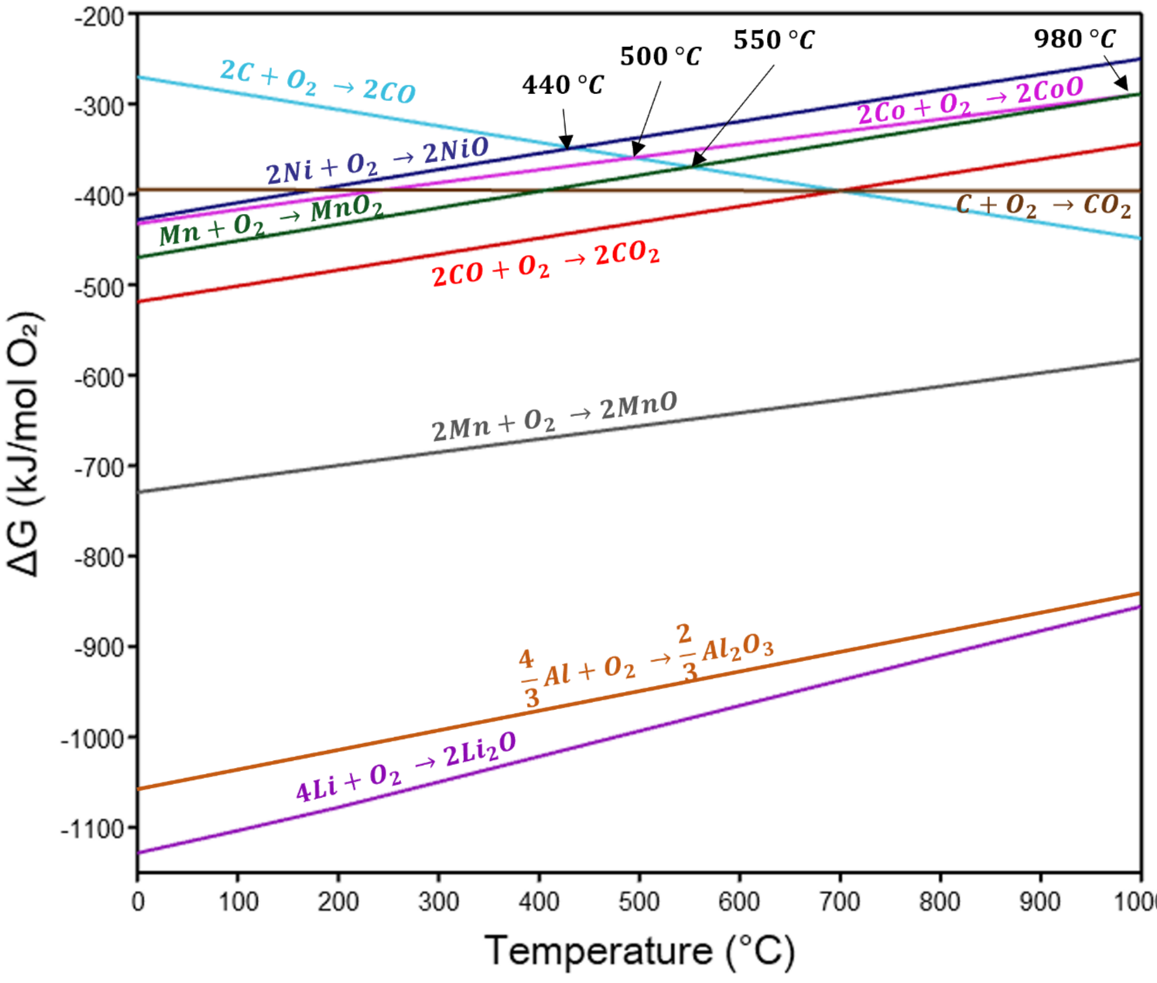

Figure S14. Ellingham diagram of lithium, nickel, manganese, cobalt, and aluminium most stable oxides elaborated with HSC Sim 10, related to Results and Discussion section. The dotted line represents the change of phase from solid to liquid.

Table S1. Detailed sections of temperature, mass change and MS ion currents of industrial black mass samples, related to Results and Discussion section.

Section		Temperature (°C)	Mass change (%)	MS main ion curves (m/z)	Compounds
I	A	50-155	-1.02	18, 28, 32 and 44	H_2O, CO, O_2 and CO_2
	B	50-166	-1.78	15, 18 and 44	EC/DEC, H_2O, and CO_2
	C	50-140	-0.68	18	H_2O
	D	50-173	-4.32	14, 15, 18, 29, 30, 44, 58 and 88	EC/DEC, NMP, H_2O, CO_2 and POF_4
	E	50-133	-0.37	18	H_2O
II	A	155-336	-2.85	18, 28, 32 and 44	H_2O, CO, O_2 and CO_2
	B	166-410	-3.78	28, and 44	CO and CO_2
	C	140-314	-2.97	18 and 44	H_2O and CO_2
	D	173-316	-1.61	14, 16, 18, 27, 29, 30, 40, 44, 64 and 85	EC/DEC, CO_2, PC, and POF_4
	E	133-406	-2.72	18, and 44	H_2O and CO_2
III	A	336-663	-4.53	18, 28, 32 and 44	H_2O, CO, O_2 and CO_2
	B	410-646	-2.70	18, 28, 32 and 44	H_2O, CO, O_2 and CO_2
	C	314-610	-8.49	14, 18, 28, 32, 44, 55, 57, 69 and 83	H_2O, CO, O_2, CO_2 and PVDF
	D	316-647	-3.09	14, 18, 29, 40, 44 and 64	H_2O, CO_2, PVDF, SO_2
	E	406-643	-2.80	18 and 44	H_2O and CO_2
IV	A	663-958	-19.83	28 and 44	CO and CO_2
	B	646-910	-19.47	28 and 44	CO and CO_2
	C	610-960	-18.54	28 and 44	CO and CO_2
	D	646-1000	-21.91	28 and 44	CO and CO_2
	E	643-983	-14.94	28 and 44	CO and CO_2
V	A	958-1000	-6.72	28	CO
	B	910-1000	-7.68	28	CO
	C	960-1000	-6.84	28	CO
	D	1000	-5.00	28	CO
	E	983-1000	-3.38	28	CO

Table S2. Detailed values of mass percentages obtained from industrial BM samples, related to Results and Discuss section.

Sample	Type of cathode	Sample mass (mg)	Initial mass loss (impurities) (%)	Mass loss CO_2/ CO (%)	New mass after impurities (mg)	Mass loss associated to CO_2/CO (mg)	Mass loss (X value) (%)	Graphite composition (Y value) (%)	Error (%)	Graphite adjusted % after Impurities (Y value)	Error (%)
A	LCO	15.77	8.40	26.46	14.45	4.17	28.89	35.29	4.87	32.57	13.91

B	LCO	17.44	8.56	26.86	15.95	4.68	29.37	34.20	3.64	28.98	13.86
C	LCO	17.76	12.16	22.52	15.60	4.00	25.64	42.57	54.25	33.17	16.79
D	NMC	16.95	9.01	26.89	15.42	4.56	29.55	32.62	6.80	28.81	21.49
E	NMC	20.44	4.05	18.36	19.62	3.75	19.13	56.37	19.94	55.91	15.94
